# Epigenetic effects of parasites and pesticides on captive and wild nestling birds

**DOI:** 10.1002/ece3.7606

**Published:** 2021-05-03

**Authors:** Sabrina M. McNew, M. Teresa Boquete, Sebastian Espinoza‐Ulloa, Jose A. Andres, Niels C. A. M. Wagemaker, Sarah A. Knutie, Christina L. Richards, Dale H. Clayton

**Affiliations:** ^1^ School of Biological Sciences University of Utah Salt Lake City UT USA; ^2^ Cornell Lab of Ornithology Cornell University Ithaca NY USA; ^3^ Department of Ecology and Evolutionary Biology Cornell University Ithaca NY USA; ^4^ Department of Integrative Biology University of South Florida Tampa FL USA; ^5^ Department of Evolutionary Ecology Estación Biológica de Doñana CSIC Sevilla Spain; ^6^ Department of Biology University of Saskatchewan Saskatoon SK Canada; ^7^ Facultad de Medicina Pontifica Universidad Católica del Ecuador Quito Ecuador; ^8^ Faculty of Science Radboud University Nijmegen The Netherlands; ^9^ Department of Ecology and Evolutionary Biology University of Connecticut Storrs CT USA; ^10^ Institute for Systems Genomics University of Connecticut Storrs CT USA

**Keywords:** DNA methylation, epiGBS, Galápagos mockingbirds, permethrin, *Philornis downsi*, pyrethroid

## Abstract

Anthropogenic changes to the environment challenge animal populations to adapt to new conditions and unique threats. While the study of adaptation has focused on genetic variation, epigenetic mechanisms may also be important. DNA methylation is sensitive to environmental stressors, such as parasites and pesticides, which may affect gene expression and phenotype. We studied the effects of an invasive ectoparasite, *Philornis downsi*, on DNA methylation of Galápagos mockingbirds (*Mimus parvulus*). We used the insecticide permethrin to manipulate *P. downsi* presence in nests of free‐living mockingbirds and tested for effects of parasitism on nestling mockingbirds using epiGBS, a reduced‐representation bisulfite sequencing (RRBS) approach. To distinguish the confounding effects of insecticide exposure, we conducted a matching experiment exposing captive nestling zebra finches (*Taeniopygia guttata*) to permethrin. We used zebra finches because they were the closest model organism to mockingbirds that we could breed in controlled conditions. We identified a limited number of differentially methylated cytosines (DMCs) in parasitized versus nonparasitized mockingbirds, but the number was not more than expected by chance. In contrast, we saw clear effects of permethrin on methylation in captive zebra finches. DMCs in zebra finches paralleled documented effects of permethrin exposure on vertebrate cellular signaling and endocrine function. Our results from captive birds indicate a role for epigenetic processes in mediating sublethal nontarget effects of pyrethroid exposure in vertebrates. Environmental conditions in the field were more variable than the laboratory, which may have made effects of both parasitism and permethrin harder to detect in mockingbirds. RRBS approaches such as epiGBS may be a cost‐effective way to characterize genome‐wide methylation profiles. However, our results indicate that ecological epigenetic studies in natural populations should consider the number of cytosines interrogated and the depth of sequencing in order to have adequate power to detect small and variable effects.

## Graphical abstract

McNew et al. studied the effects of parasites and permethrin exposure on DNA methylation of nestling birds. They found that permethrin exposure in captivity results in differences in methylation; however, the same differences were not seen in wild birds, in response to either parasitism or permethrin.

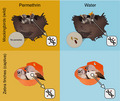

## 1 INTRODUCTION

Invasive parasites and pathogens pose grave threats to wildlife populations (Hoyt et al., 2020; Scheele et al., 2019), as well as parallel opportunities to study the mechanisms and evolution of host defense (Bonneaud et al., 2011; Jones et al., 2008). Genetic variation is thought to underlie differences in susceptibility to disease (Archie et al., 2009; Fumagalli et al., 2011). A population that survives the introduction of a virulent parasite or pathogen must either have standing genetic variants that confer protection, or novel mutations must occur and spread through the population. However, recent work has identified other molecular mechanisms that can generate a rapid response to an emerging threat. For example, changes in gene expression related to resistance are one way in which animals can adapt to a new parasite (Bonneaud et al., 2011; Navajas et al., 2008). Thus, rapid responses to new stressors may be mediated by changes in expression of existing genes and pathways.

Although changes in gene expression have been linked to host defense against parasitism, the processes that regulate expression are complex and we know little about how those processes are affected by the environment in natural systems. One candidate regulatory mechanism is DNA methylation, the binding of methyl groups to nucleotides, typically cytosines (Angers et al., 2010). Changes in DNA methylation can also affect gene expression and the resulting phenotype without changing the DNA sequence itself (Duncan et al., 2014; Jaenisch & Bird, 2003; Jones, 2012; Robertson, 2005). Studies in human and other animal systems demonstrate that methylation profiles are sensitive to exposure to toxicants or stressors early in development (Baccarelli & Bollati, 2009; Onishchenko et al., 2008). Moreover, some methylation changes persist in the germ line, meaning the phenotypes they encode could be passed on to subsequent generations, providing a mechanism for rapid evolution in response to environmental change (Crews et al., 2007; Janowitz Koch et al., 2016; Latzel et al., 2012; McNew et al., 2017; Ortega‐Recalde & Hore, 2019; Richards & Pigliucci, 2020; Skinner et al., 2010; Verhoeven et al., 2016).

DNA methylation has been associated with adaptation to various environmental changes and stressors in wild animals (Artemov et al., 2017; Heckwolf et al., 2020; Hu et al., 2019; Mäkinen et al., 2019; McNew et al., 2017; Rubenstein et al., 2016; Taff et al., 2019; Watson et al., 2021; Weyrich et al., 2016). For example, in a study of superb starlings (*Lamprotornis superbus*), Rubenstein et al. (2016) found associations between rainfall and DNA methylation in the glucocorticoid receptor. In this study, DNA methylation levels were also linked to the reproductive success of male starlings, suggesting that methylation changes can mediate an adaptive response to environmental conditions early in life. DNA methylation changes have also been associated with effects of parasites on vertebrate hosts (Hu et al., 2018; Wenzel & Piertney, 2014). In a study of red grouse (*Lagopus lagopus scotica*) populations, Wenzel and Piertney (2014) found associations between nematode parasite load and methylation at a subset of loci using methylation‐sensitive AFLP (MSAP) markers. A different study using reduced‐representation bisulfite sequencing (RRBS) found genome‐wide effects associated with ectoparasites on guppy (*Poecilia reticulata*) methylation, including several areas that were associated immune‐related proteins (Hu et al., 2018). These studies provide evidence that epigenetic changes may help individuals flexibly respond to natural stressors, including parasites and pathogens. However, the degree to which epigenetic mechanisms are involved in host responses to emerging disease is still largely unknown (Gómez‐Díaz et al., 2012; Poulin & Thomas, 2008).

In this study, we tested for epigenetic effects of an introduced parasitic nest fly, *Philornis downsi*, on Galápagos mockingbirds (*Mimus parvulus*). *Philornis downsi* is one of the most significant threats to endemic Galápagos land birds (Causton et al., 2006; McNew & Clayton, 2018; O’Connor et al., 2010). Adult *P. downsi* flies are free‐living; however, the larval stages live in the nests of birds and feed on brooding mothers and their nestlings. *Philornis downsi* causes high nestling mortality in many species of Galápagos passerines, and parasitism has been associated with population declines of some species of Darwin's finches (Fessl et al., 2010; Koop et al., 2016; McNew et al., 2019; O’Connor et al., 2010b). Galápagos mockingbirds show variable tolerance to *P. downsi*; in years of high food abundance, most parasitized nestlings survive, but in drought years, parasitism causes high mortality (Knutie et al., 2016; McNew et al., 2019). However, in all years parasitism causes significant blood loss and decreases hemoglobin concentration in nestlings (McNew et al., 2019). Parasitism affects the expression of genes related to metabolism, signaling, and transcription in mockingbirds, which could reflect both the costs of *P. downsi* parasitism and potential mechanisms of tolerance (Knutie, 2014).

**FIGURE 1:**
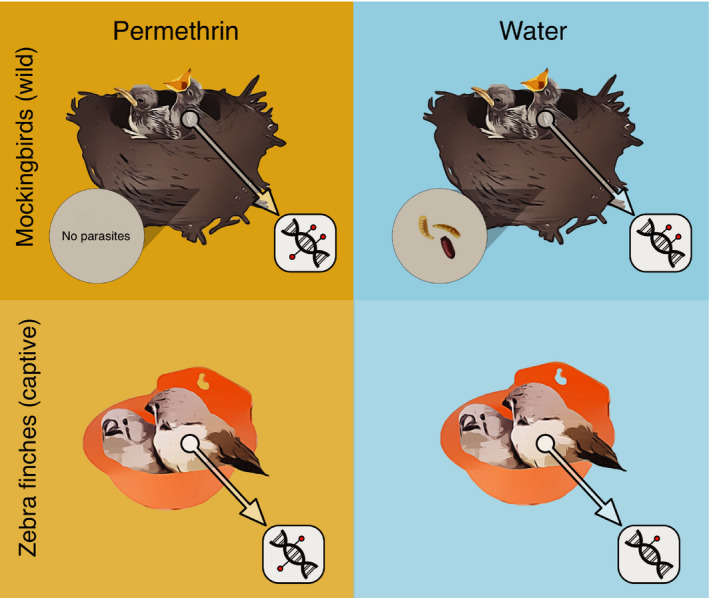
Schematic illustrating experimental design. We experimentally manipulated *P. downsi* in the nests of free‐living mockingbirds to test for epigenetic effects of parasitism. Nests were treated with permethrin, to exclude parasites, or water, as a control. To examine the effects of permethrin, we sprayed zebra finch nests in captivity with permethrin or water. We sequenced one nestling per nest of each species (*N* = 21 mockingbirds; *N* = 11 zebra finches). Nests and nestlings are not shown to scale; mockingbird nestlings weigh about 3.5 g at hatching; zebra finch nestlings weigh about 0.75 g (unpublished data). Mockingbirds construct large nests of sticks, moss, and grass; captive zebra finches were supplied with plastic wall‐mounted nest cups and shredded newspaper for nest material. Modified from an illustration by Jennifer Lobo

To further investigate the molecular effects of *P. downsi* on mockingbirds, we experimentally manipulated the presence of *P. downsi* in mockingbird nests and characterized the DNA methylation profiles of mockingbird nestlings (Figure 1). We used epiGBS, a reduced‐representation sequencing method to identify DNA methylation (van Gurp et al., 2016). epiGBS combines the use of a restriction enzyme to sample the genome with bisulfite sequencing to identify methylated cytosines. It is an efficient and cost‐effective way of characterizing genome‐wide methylation profiles with single‐nucleotide resolution. First, we tested whether global methylation profiles differed between treatments and/or whether specific nucleotides were differentially methylated between treatments. Second, we investigated whether any methylation changes occurred near genes with a putative functional relationship to parasitism, such as immune or inflammatory genes, genes involved in erythrocytosis, or genes that were previously identified as differentially expressed in response to parasitism (Knutie, 2014).

Because we used permethrin, a pyrethroid insecticide, to experimentally manipulate *P. downsi*, we complemented the field study with a parallel experiment of the effects of permethrin exposure on nestling DNA methylation. Permethrin is a synthetic insecticide in the pyrethroid family, which was developed from compounds derived from chrysanthemum flowers (Casida, 1980; Perveen, 2011). Pyrethroids affect the nervous system of insects by affecting sodium channels and therefore nerve cell impulse transmission (Perveen, 2011; Sattelle & Yamamoto, 1988). Pyrethroids became one of the most broadly used classes of insecticides in part because they have low toxicity in most mammals and birds (Casida, 1980; Perveen, 2011). Although permethrin is largely considered “safe” for birds, it may have sublethal effects on nestling growth and physiology (Bulgarella et al., 2020; Hund et al., 2015; López‐Arrabé et al., 2014). The use of pyrethroids in the Galápagos has therefore been subject to scrutiny due to concern about nontarget effects on nestlings and native arthropods (Bulgarella et al., 2020; Causton & Lincango, 2014). Breeding Galápagos mockingbirds in captivity away from *P. downsi* was not possible, so instead we used captive zebra finches (*Taenopygia guttata*), exposing nestlings to permethrin using the same methods as in the field (Figure 1). We compared the results of mockingbirds and zebra finches to disentangle the epigenetic effects of the insecticide from the effects of parasitism.

## 2 METHODS

### 2.1 Experimental manipulation of *P. downsi*

Field experiments took place at El Garrapatero (0°41′23.3″S, 90°13′21.0″W), a 3 × 4 km coastal scrub site on Santa Cruz Island, during part of a broader study of the effects of *P. downsi* on mockingbird reproductive success (Knutie et al., 2016; McNew et al., 2019). Mockingbird nests were identified during the beginning of the breeding season in 2012, 2013, 2015, and 2016. Soon after the chicks hatched, we briefly removed them while spraying the nests with either 1% aqueous permethrin (Permectrin™ II; Bayer) or water as a control, following previously used methods (Fessl et al., 2006; Knutie et al., 2016; Koop et al., 2013). While treating the nest, we removed the top layer of nesting material and sprayed the interior with a generic spray bottle with a fine mist setting of the nest where *P. downsi* typically reside. The nest was sprayed until the interior was damp to the touch. After the nest dried (<10 min), the top layer of the nest and the nestlings were replaced. We sprayed nests again at 5–6 days after hatching (Knutie et al., 2016; McNew et al., 2019). Permethrin is commonly used to eliminate *P. downsi* in the Galápagos and has not been associated with any detectable negative effects on native birds (Causton & Lincango, 2014; Kleindorfer & Dudaniec, 2016; Koop et al., 2013b).

We quantified *P. downsi* by collecting each nest after the nestlings either fledged (at about 15 days) or died. We dissected nests within 8 hr and carefully counted *P. downsi* larvae and pupae. The treatment method does not completely eliminate exposure to parasites because nestlings in the fumigation treatment could have been parasitized during the first few hours after hatching. However, once nests are treated, permethrin is extremely effective at eliminating *P. downsi* (Koop et al., 2013b; McNew et al., 2019). When nestlings were 9–11 days old, we weighed and measured each nestling, and collected a small blood sample from each nestling via brachial venipuncture. Blood samples were stored on wet ice in the field. Within 6 hr of collection, the blood samples were centrifuged at 7,800 *g* for 10 min to separate plasma and erythrocytes, which were frozen separately (not in buffer) in a −20°C freezer. Samples were transported to the University of Utah in a liquid nitrogen dry shipper, where they were permanently stored in a −80°C freezer. In total, we followed 126 nests over all 4 years. We haphazardly selected one nestling per nest for sequencing, balancing treatments and years. Sampling was limited by the number of blood samples in 2015, a year in which most parasitized nestlings died before blood collection. We had samples from nestlings in nine different parasitized nests that year; thus, we elected to sequence nine nestlings per treatment (permethrin/nonparasitized, water/parasitized) in each of the 4 years (total *N* = 72; Table S1).

### 2.2 Side effects of permethrin exposure

We tested for effects of permethrin exposure on nestling DNA methylation using zebra finches because they were the most closely related bird species to mockingbirds with a well‐annotated genome that we could breed in a controlled environment. We bred zebra finches in an indoor aviary at the University of Utah where they had access to nesting material (shredded paper) and wall‐mounted plastic nest cups. When chicks hatched, they were briefly removed while we treated the nest with either permethrin or water. We shifted the nesting material aside to spray the interior and base of the nest in a similar way to the mockingbird nests. Due to the loose and simple construction of the nests, it was impossible to completely avoid spraying the surface of the nest, where the nestlings sat. As a result, the nestlings may have come into contact with the treatment, even after it dried. We sprayed nests again 7 days after hatching (the nestling period for zebra finches is longer than that for mockingbirds: 21 vs. 14 days, respectively). At 15 days after hatching, we took blood samples from nestlings via brachial venipuncture. Blood samples were centrifuged to separate plasma and erythrocytes, and erythrocytes were frozen in TRIzol™ Reagent (Life Technologies). In total, we treated 11 nests (six permethrin and five water); one nestling from each was randomly selected for sequencing (Table S1).

### 2.3 Validation of zebra finches as a model for Galápagos mockingbirds

Zebra finches and Galápagos mockingbirds are from different avian families and native to different continents. However, the overall structure, organization, and size of passerine genomes are highly conserved (Ellegren, 2013; Hooper & Price, 2017). We tested whether our two species had similar genomic structure and size and used an in silico digestion to determine whether the epiGBS protocol would yield comparable results between species. First, we compared the genome structure of mockingbirds and zebra finches using the reference genome for each: zebra finch reference genome v.bTaeGut2.pat.W.v2 downloaded from NCBI (Koepfli et al., 2015) and the San Cristobal mockingbird (*M. melanotis*) reference genome, a congener and a close relative of the Galápagos mockingbird (S. E. Ulloa and J. A. Andres, unpublished data). The chromosome sizes were remarkably similar and highly correlated between species (*r* = .99; Figure S1). Next, we used the program FRAGMATIC (Chafin et al., 2018) to digest each genome and create fasta files of fragments 200–800 bp long, following the expected size distribution of the epiGBS protocol (Boquete et al., 2020). We aligned the fragments to the reference genome of the other species (i.e., aligned zebra finch fragments to the mockingbird reference genome and vice versa) using bwa (Li, 2013) to determine whether the restriction enzyme would produce fragments present in both species. The digestion produced ~266 thousand fragments from the mockingbird genome, including 1,057,346 CpG loci and ~279 thousand fragments from the zebra finch genome, including 1,158,380 CpG loci. For mockingbirds, 95.5% of fragments aligned to the zebra finch genome. For zebra finches, 92.5% of fragments aligned to the mockingbird genome. These results demonstrate that there is high synteny between species and that our sequencing method targets segments present in both genomes. The commands used for the in silico digestion are available in the insilico_digest.sh script (https://github.com/smcnew/epigbs).

### 2.4 DNA extraction

Genomic mockingbird DNA was extracted from frozen erythrocytes using DNeasy kits (Qiagen, Cat. #69506) following the manufacturer's protocols. DNA from zebra finches was isolated from TRIzol‐preserved samples using the following chloroform protocol: Samples were first incubated in 1 ml TRIzol for 5 min at room temperature. Next, 200 µl chloroform was added and samples were incubated for 15 min at room temperature. Samples were then centrifuged at 50,000 *g* for 15 min at 8°C. The supernatant was discarded, and then, the DNA was precipitated in 100% ethanol. After 3‐min incubation at room temperature, the sample was centrifuged for 5 min at 14,000 RCF. The supernatant was again discarded, and the DNA pellet was washed twice in 1 ml 0.1 M sodium citrate. DNA was resuspended in 75% ethanol, followed by 5 min of centrifugation at 14,000 RCF. The supernatant was removed and the pellet was dried for 5 min on the bench. The DNA pellet was dissolved in 100 µl sterile water and incubated at 55°C for 10 min. Samples were then centrifuged for 10 min at 18,000 *g*, and the supernatant was removed and placed in a new tube. DNA concentration from extracted DNA samples was quantified using 5 μl of the extraction and a Qubit 4 Fluorometer (Invitrogen).

### 2.5 Library preparation and sequencing

We used epiGBS, a RRBS method to identify DNA methylation (Boquete et al., 2020; van Gurp et al., 2016). Briefly, 400 ng of DNA was digested for 17 hr at 37°C in a 40 μl reaction containing the restriction enzyme PstI, NEBuffer 3.1 (New England Biolabs [NEB]; Ipswitch, MA), and BSA (NEB). Unique index adapter pairs were ligated onto each sample to enable sample identification after pooling. Barcodes (between 4 and 6 bp long) were paired in combinations so that each pair of barcodes differed from all others by a minimum of three mutational steps (Table S1). Adaptor ligation took place in a 60 μl reaction containing 40 μl of digested DNA, along with 6 μl T4 DNA ligase buffer, 1 μl T4 DNA ligase (NEB), and 2.4 ng of each barcoded adaptors. Reactions were run for 3 hr at 22°C and then overnight at 4°C on a Techne TC‐4000 thermocycler.

Samples were pooled in equal final concentrations in groups of eight, after which we did a PCR cleanup step using a QIAquick PCR Purification Kit (Qiagen) following the manufacturer's protocol. The libraries were size selected using 0.8× SPRI beads (Magbio) to purify fragments approximately 200–800 bp long, which were eluted in a total volume of 24 μl (Boquete et al., 2020). Libraries were nick‐repaired to correct gaps between the barcode adaptors and DNA fragments. Nick repair took place in a 1‐hr reaction at 15°C containing 18 μl of the purified DNA, 2.5 μl dNTPs containing 5‐methylcytosine (5mC; Zymo Research), 2.5 μl 10× NEB buffer 2 (NEB), and 0.75 μl DNA polymerase I (NEB).

We bisulfite‐converted the libraries using the EZ DNA Methylation‐Lightning Kit (Zymo Research) following the manufacturer's protocol. Immediately following bisulfite conversion and cleanup, we amplified the libraries with PCR. Reactions included 5 μl of KAPA HiFi HotStart Uracil + ReadyMix (Kapa Biosystems) and 6 pmol of primers (van Gurp et al., 2016). PCR cycles involved an initial denaturation step for 3 min at 95°C followed by 18 cycles of 98°C for 10 s, 65°C for 15 s, and 72°C for 15 s, with a final extension step at 72°C for 5 min. Libraries were bioanalyzed using 1 μl of DNA on a high‐sensitivity DNA chip on a 2100 Bioanalyzer (Agilent Technologies). Libraries were pooled in equimolar quantities (using 0.2–4.3 μl per library) into a single pool containing approximately 200 ng of DNA. The library was then sequenced in a single lane on an Illumina Hiseq PE150 sequencer at Novogene Co. (Hong Kong).

### 2.6 Quality assessment, alignment, and variant calling

The quality of sequencing reads was characterized using FastQC v.0.11.4, after which low‐quality bases and adapters were trimmed using TrimGalore! v.0.4.4 using default parameters. Demultiplexing was done using Python scripts developed for epiGBS (van Gurp et al., 2016) and modified for this project (demultiplex_MHR.py; https://github.com/smcnew/epigbs). Reads from each individual were then sorted into individual forward and reverse fastq files using the standard “grep” command to search individual sample names (grep_individuals.sh; https://github.com/smcnew/epigbs). We used bwameth v0.2.2 with default parameters (Pedersen et al., 2014) to align reads for each mockingbird to the San Cristobal mockingbird (*Mimus melanotis*) reference genome (S.E. Ulloa and J. A. Andres, unpublished data). Reads for each zebra finch were aligned to the zebra finch (*Taeniopygia guttata*) reference genome (v. bTaeGut2.pat.W.v2) downloaded from NCBI (Koepfli et al., 2015).

Methylation polymorphisms were called using methyldackel (https://github.com/dpryan79/MethylDackel; v.0.3.0) with the following procedure: First, individual plots of methylation bias along reads were made using the mbias function (Figure S2). Methylation bias occurs when methylation levels vary predictably along the position of a read and is common at the beginnings and ends of reads (Mäkinen et al., 2019). Using visual inspection of mbias plots and suggested bounds from methyldackel, we trimmed between 10 and 50 bp from the beginning and end of reads of each strand for each individual in order to exclude biased regions using the methyldackel options ‐‐nOT, ‐‐nOB, ‐‐nCTOT, and ‐‐nCTOB (exact commands available in gamo_analysis_final.sh; https://github.com/smcnew/epigbs). We then called variants using the ‘extract’ command including duplicate reads (‐‐keepDupes) because epiGBS is an enrichment‐based library preparation and some duplicates are expected (Krueger & Andrews, 2012). We limited our analysis of methylation to cytosines followed by guanines (“CpG” sites) because non‐CpG methylation is negligible in birds (Mäkinen et al., 2019).

### 2.7 Identification and annotation of differentially methylated cytosines

Individual differentially methylated cytosines (DMCs) between treatment groups were identified using R (v3.5.2) in the RStudio environment (v1.3.1093) and the package methylKit (v1.12.0; Akalin et al., 2012). For each individual, we filtered out cytosines with coverage <10 reads and those with coverage greater than the 99.9th percentile of coverage for each sample using the filterByCoverage command. After filtering based on read coverage, many mockingbirds had very few sequenced cytosines. We removed low coverage individuals (with less than 20,000 sequenced cytosines; see Figure S3), which resulted in 21 mockingbirds in the final dataset (Table S2). No zebra finch individuals were excluded from the final dataset (all had at least 20,000 sequenced cytosines; *N* = 11). Then, for each species, we normalized coverage among individuals using the normalizeCoverage command. We ran a PCA using the PCASamples function in methylKit to investigate whether overall methylation profiles differed between treatments within each species. Finally, we tested for differential methylation between treatments at each cytosine using a logistic regression model using the calculateDiffMeth command. The model for zebra finches included only the effect of treatment (permethrin or water). The model for mockingbirds included the covariate of year and treatment (permethrin/nonparasitized or water/parasitized). Only those cytosines that were sequenced in at least five individuals per treatment for mockingbirds and four individuals per treatment for zebra finches were included. P values were adjusted for multiple comparisons using the SLIM method, creating “*q* values” (Wang et al., 2011). Cytosines that had a *q* value <0.01 and a difference in methylation levels between groups of at least 25% were considered significantly differentially methylated (Akalin et al., 2012; Hu et al., 2018; Watson et al., 2021).

The location of DMCs with respect to genomic features was determined with the genomation R package v1.18.0 using the *M*. *melanotis* genome assembly and annotation (Ulloa and Andres unpublished data) for mockingbirds and the zebra finch NCBI Refseq annotation. We determined whether DMCs mapped to exons, introns, promoters (i.e., within 2kb of a transcriptional start site), or intergenic regions. Overlapping features were assigned hierarchically with promoter > exon > intron precedence. We investigated gene ontology (GO) categories associated with DMCs using the PANTHER classification system hosted on the Gene Ontology Consortium (Ashburner et al., 2000; The Gene Ontology Consortium, 2019; geneontology.org; release 20200728). We compared our gene set against the *Gallus gallus* gene database (https://doi.org/10.5281/zenodo.4081749 release date 2020‐10‐09). We used Fisher's exact test to test for overrepresentation of biological functions among the genes associated with DMCs (including DMCs in promoter, exons, or introns of the gene). Then, we conducted separate GO analyses of genes with DMCs in promoters and genes with DMCs in exons, as methylation in those regions may be particularly important for regulating expression (de Mendoza et al., 2020; Schmitz et al., 2019).

We visualized biological processes using the hierarchical clustering function on the REVIGO database, which merges semantically similar GO categories (Supek et al., 2011). We included all GO terms with a *p* value < .01 identified by PANTHER, clustering terms with a user‐specified similarity of 0.5. Finally, we compared the list of genes associated with sequenced cytosines and DMCs in mockingbirds with those previously identified as differentially expressed in mockingbirds parasitized by *P. downsi* (Knutie, 2014).

### 2.8 Controls for false positives

We controlled for potential false positives and artifacts in our results in two ways: First, after initial filtering and quality control steps, we ran a simulation that randomly assigned treatments to individual birds and reran the logistic regressions to determine how many DMCs we would expect by chance for each species. We created a null distribution of 1,000 simulations for both mockingbirds and zebra finches and compared our observed results with the expected distribution. Second, because we sequenced more individuals in our mockingbird dataset than our finch dataset, we also investigated the sensitivity of our results by increasing the required minimum number of individuals sequenced at a cytosine in order for that position to be analyzed. We report the number of DMCs found when analyzing only those cytosines sequenced in at least 6, 8, and 10 individuals per treatment. Our hypothesis was that including more individuals per treatment would reduce the number of cytosines analyzed and therefore produce fewer DMCs. However, we expected that the proportion of observed‐to‐expected DMCs would stay consistent no matter the cutoff number. This outcome would indicate that the cutoff value of individuals per group does not significantly affect the results.

## 3 RESULTS

### 3.1 Mockingbird methylation results

**FIGURE 2:**
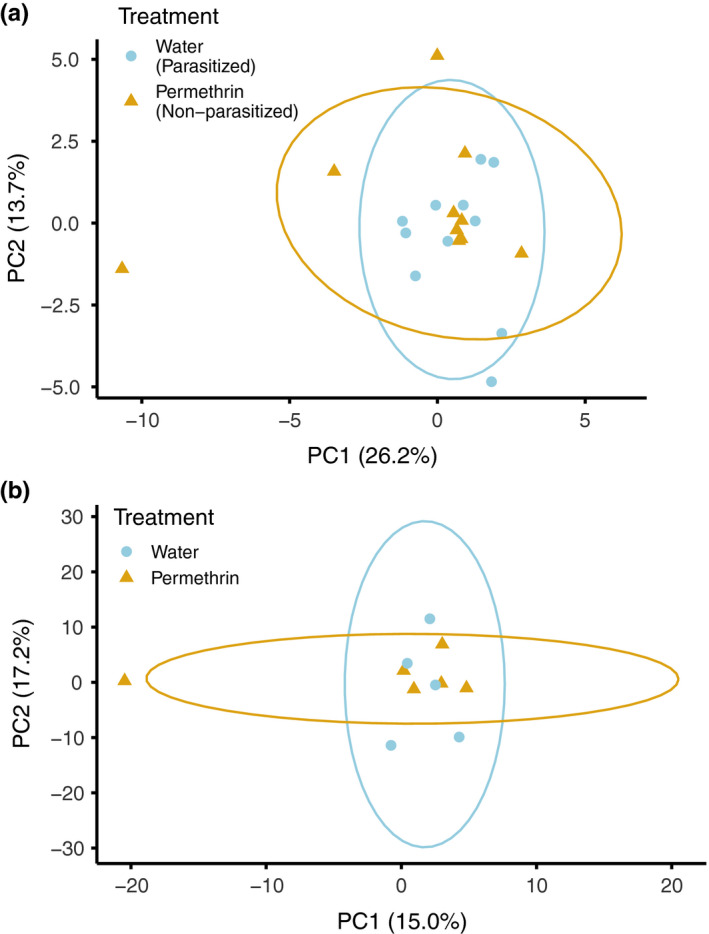
Principal components analysis of methylation profiles based on 11,419 cytosines for mockingbirds (a) and 1,238 cytosines for zebra finches (b). Each point is an individual; colored symbols indicate treatment

Our final dataset included 11 permethrin‐treated mockingbirds (i.e., nonparasitized) and 10 control mockingbirds (i.e., parasitized). Permethrin treatment significantly reduced *P. downsi* abundance; the number of *P. downsi* found in permethrin‐treated nests was 0.6 ± 0.42 SE; for control nests, the mean *P. downsi* was 68.6 ± 19.7 (negative binomial generalized linear model, *p* <.001; Table S2). After preliminary trimming and filtering steps, we aligned a mean of 10,901,272 reads per individual to the reference genome (range: 1,950,039–24,053,910; Table S2). The mean alignment rate to the reference genome was 99.47% (range: 99.25%–99.70%). We recovered between 23,303 and 196,141 cytosines sequenced to at least 10× coverage per individual mockingbird of which 11,419 cytosines met the criteria for differential methylation analysis (i.e., sequenced for at least five individuals per treatment). Cytosines were distributed fairly evenly across all autosomes and the Z chromosome (Figure S4). Across all positions, the mean methylation percentage was 62.0% (standard deviation = 27.5%). PCA did not show any differentiation between treatments in overall methylation profile (Figure 2a). However, 194 (1.7%) individual cytosines were significantly differentially methylated between treatments (“DMCs”; Figure S4, Table S3).

**FIGURE 3:**
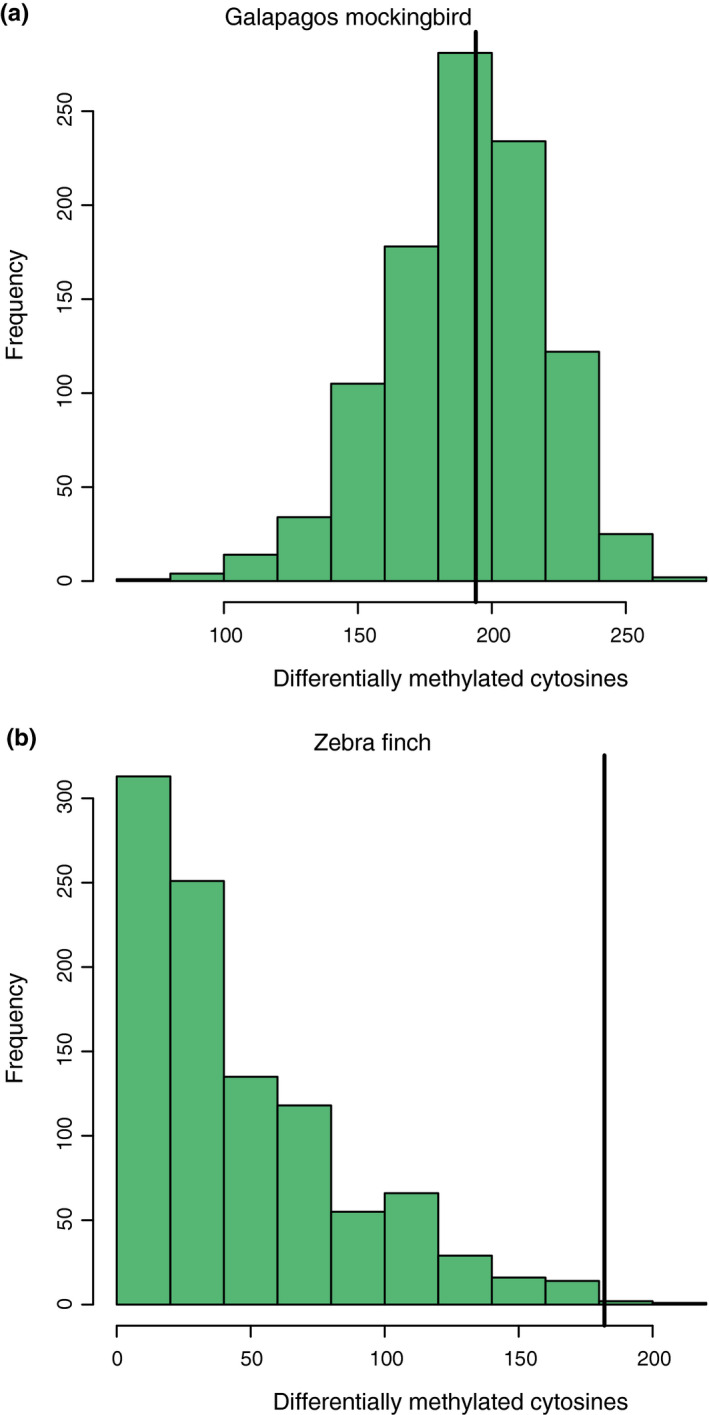
Distribution of expected numbers of differentially methylated cytosines (DMCs) for mockingbirds (a) and zebra finches (b) based on randomizing individuals with respect to treatment. Results are from 1,000 simulations for each dataset (mockingbird median = 193; zebra finch median = 34). The black vertical line in each panel represents the observed number of DMCs between true treatment groups (mockingbird = 194; zebra finch = 182)

We randomized treatments of mockingbirds and calculated the number of DMCs observed between two arbitrary groups of individuals. After 1,000 simulations, we recovered a normal distribution of DMCs where the median was 193, one fewer than we found between our true treatment groups (194; Figure 3). Increasing the number of individuals per treatment group required for a cytosine to be tested reduced the number of DMCs detected; however, in all cases, the number detected was very similar to the median of a simulated null distribution (Table 1).

**TABLE 1:** CpGs and DMCs recovered after increasing the minimum number of mockingbirds per treatment for a cytosine to be tested

Samples per treatment	CpGs	Observed DMCs	Expected DMCs^a^
5^b^	11,419	194	193
6	4,460	37	41.5
8	524	2	1
10	88	0	0

^a^ Expected numbers for *N* = 6–10 based on the median number observed in 100 simulations of individuals randomized to treatment.

^b^ Results in text are based on *N* = 5.

**FIGURE 4:**
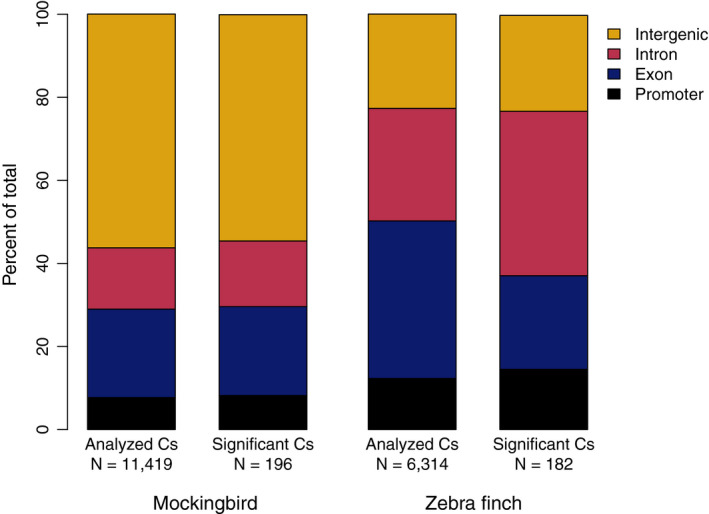
Distribution of analyzed cytosines and significantly differentially methylated cytosines among genomic features for each species

**FIGURE 5:**
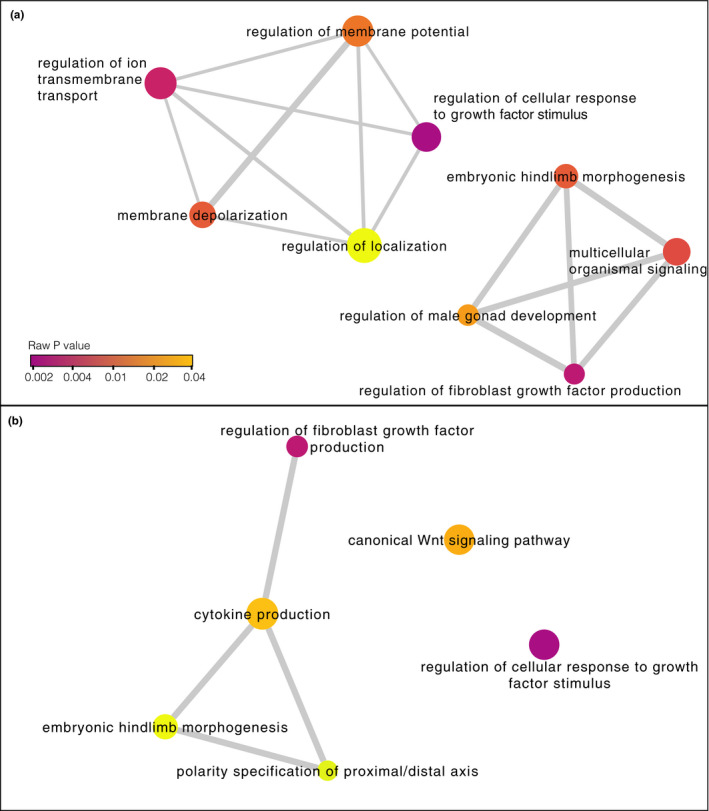
Network illustrating the biological functions of genes associated with mockingbird DMCs. (a) All genes overlapping DMCs (including DMCs in exons, introns, or promoters); (b) only those genes with DMCs in promoters. For each network, we identified biological functions that were overrepresented compared with the entire GO Annotation database (raw *p* value < .001). Then, we clustered and visualized those terms in REVIGO (Supek et al., 2011). The color of the node is proportional to the *p* value; darker colors indicate smaller *p* values. The size of the node indicates the frequency of the term in the GO Annotation database (nodes of more general terms are larger). Similar GO terms are linked by network edges; the line width within each panel indicates the degree of similarity. Position of some node labels was adjusted for readability

Of the 194 DMCs found between true treatment groups, 87 were associated with genes (66 genes in total; Figure 4, Tables S3 and S4). We further categorized those DMCs by genomic feature: 16 DMCs mapped to promoters (10 genes); 43 DMCs mapped to exons (32 genes); and 31 DMCs mapped to introns (25 genes). Gene ontology (GO) analysis using PANTHER did not detect any significantly overrepresented biological processes associated with DMCs in promoters, exons, or the entire gene set combined (including DMCs in exons, promoters, and introns; FDR for all categories >0.01). We used REVIGO to cluster GO terms with a *p* value < .001 for all genes associated with DMCs (Figure 5a) and for genes associated with DMCs in promoters (Figure 5b). There were no GO terms with *p* < .001 for any genes with DMCs in exons. GO terms clustered into several biological processes including regulation of cellular response to growth factor stimulus and cytokine production.

We tested whether any of the genes associated with sequenced cytosines overlapped with those 46 genes that were differentially expressed (raw *p* value ≤ .01) in response to *P. downsi* (Knutie, 2014). The cytosines sequenced in our mockingbird dataset (including both differentially methylated and nondifferentially methylated cytosines) mapped to the introns, exons, or promoter regions of 1,797 genes. However, there was no overlap among these genes and the genes differentially expressed in response to *P. downsi* (Knutie, 2014). Correspondingly, there was also no overlap between the genes associated with DMCs and the differentially expressed genes (sensu Alvarez et al., 2020).

### 3.2 Zebra finch methylation results

We analyzed methylation patterns of six treated and five control zebra finches. Following initial trimming and filtering, we aligned a mean of 7,846,499 reads per individual to the zebra finch reference genome (range: 3,453,809–18,511,302; Table S2). Mean alignment rate was 99.51% (range: 99.27%–99.69%). After variant calling, our dataset included between 21,242 and 127,794 cytosines per individual, of which 1,238 cytosines across the genome were tested for differential methylation. Across all positions, the mean methylation percentage was 69.5% (standard deviation = 27.4%). Sequenced cytosines most frequently mapped to exons (43.0%), with the remainder distributed across promoters, introns, and intergenic regions (Figure 4). Again, a PCA of methylation polymorphisms revealed no overall differentiation in methylation profiles between treatments (Figure 2b). Out of the tested cytosines, 182 (14.7%) were significantly differentially methylated (Figure S4, Table S5).

We randomly assigned zebra finches to treatment and calculated the number of DMCs observed between two arbitrary treatment groups. We recovered a right‐skewed distribution of DMCs where the median number of DMCs observed was 34 (Figure 3b). Only three out of 1,000 simulations produced DMCs equal to or greater than the number we observed between the true treatment groups (182), indicating that we observed more DMCs between permethrin and water treatments than expected by chance (*p* = .003).

**FIGURE 6:**
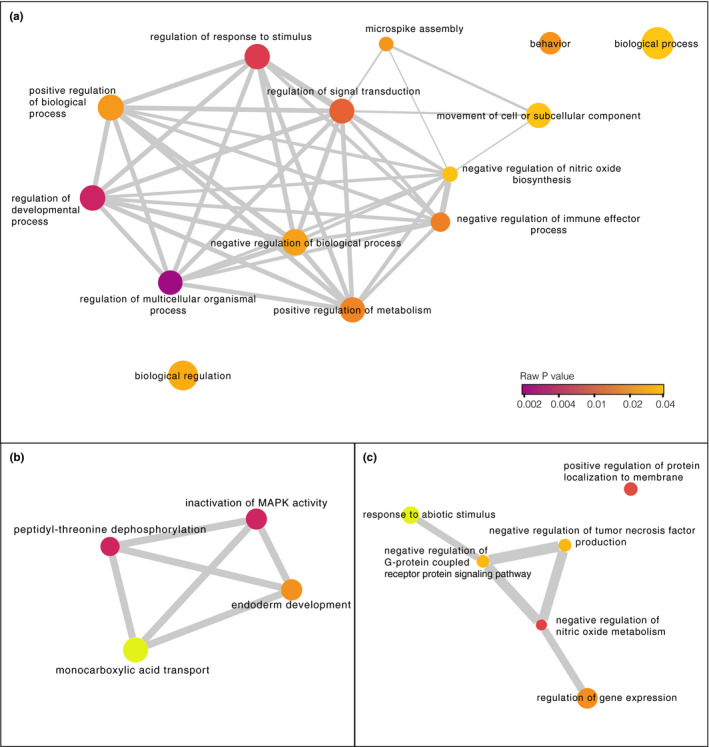
Network illustrating the biological functions of genes associated with zebra finch DMCs. (a) All genes overlapping DMCs (including DMCs in exons, introns, or promoters); (b) only those genes with DMCs in promoters; (c) only those genes with DMCs in exons. For each network, we identified biological functions that were overrepresented compared with the entire GO Annotation database (raw *p* value < .001). Then, we clustered and visualized those terms in REVIGO (Supek et al., 2011). The color of the node is proportional to the *p* value; darker colors indicate smaller *p* values. The size of the node indicates the frequency of the term in the GO Annotation database (nodes of more general terms are larger). Similar GO terms are linked by network edges; the line width within each panel indicates the degree of similarity. Position of some node labels was adjusted for readability

The DMCs mapped to the promoter regions of 20 genes (27 DMCs) and to the exons of 45 genes (46 DMCs), and were associated with a total of 122 genes (including 140 DMCs in promoters, exons, and introns; Figure 4; Table S4). There was no overlap in the genes associated with DMCs in zebra finches and genes identified in the mockingbird analysis. GO analysis did not reveal any categories that were significantly overrepresented (FDR for all >0.01). REVIGO clustering of GO terms with a *p* < .001 identified biological functions including monocarboxylic acid transport, negative regulation of nitric oxide metabolism, and regulation of biological processes (Figure 6).

## 4 DISCUSSION

We tested whether *P. downsi* affected DNA methylation of Galápagos mockingbirds, assessing the effects of the insecticide used to manipulate parasitism through a separate experiment with captive zebra finches. Although we did identify more than one hundred DMCs that were significantly associated with parasitism in mockingbirds, this number was nearly identical to the number expected by chance. In contrast, we identified far more DMCs than expected by chance between zebra finch treatment groups. These results suggest that permethrin does have epigenetic effects on developing nestlings, but the effects may be either specific to exposure in captive conditions or species‐specific. We expected our sequencing method to target similar genomic regions in our two study species since their genomes are very similar in size and structure and in silico digestion‐produced regions present in both genomes. Nevertheless, as with other RRBS studies (Paun et al., 2019), our actual genome coverage was sparse, revealing limitations in our ability to detect differences between treatments.

### 4.1 Effects of *P. downsi* on mockingbird methylation

The DMCs we found were associated with genes linked to a variety of biological processes (Figure 5). Several of these processes relate to cell signaling and development, including the regulation of cellular response to growth factor stimulus and regulation of fibroblast growth factor production (Figure 5b). *Philornis downsi* has negative effects on nestling development (McNew et al., 2019). Particularly in drought years when food is limited, parasitism slows the rate of nestling skeletal growth and feather emergence (McNew et al., 2019). The expression of fibroblast growth factors contributes to feather development (Song et al., 1996). Thus, developmental delays caused by parasitism could conceivably be reflected in epigenetic changes to these signaling pathways. However, we are cautious in our interpretation of these functional effects because the number of DMCs we observed was nearly identical to the number expected between two random groups of nestlings. Thus, it is possible the differences we found occurred purely by chance and do not reflect the effects of *P. downsi*.

Epigenetic responses of hosts to ectoparasitism are likely to be complex, since the parasites may damage several tissues and areas of the body. For example, methylation patterns of captive Trinidadian guppies (*Poecilia reticulata*) were altered in dynamic ways over the course of infection by a monogenean ectoparasite (Hu et al., 2018). In that study, differentially methylated genes spanned a range of biological functions, from genes involved in wound healing and skin development to genes involved in immune defense. Here, we found differential methylation in the promoter of the gene WNT8C (Figure 5b), which could be related to innate immune function through effects of Wnt signaling on inflammation and cytokine production (Oderup et al., 2013). Although nestling mockingbirds do not mount a detectable antibody response to *P. downsi* (Knutie et al., 2016), little is known about whether *P. downsi* activates aspects of the innate immune response. Innate immune defenses are expected to develop faster than adaptive defenses (Palacios et al., 2009) and thus could be important for juvenile hosts responding to parasitism. Future studies could investigate whether *P. downsi* induces innate immune components in nestlings, including cytokine production, inflammation, and the production of monocytes and heterophils.

We did not observe any overlap between the genes associated with differential methylation and those differentially expressed in response to parasitism (Knutie, 2014). However, the two studies used very different approaches (epiGBS here vs. microarrays in Knutie, 2014) and different reference genomes (Floreana mockingbird here vs. the zebra finch genome in the earlier study). The cytosines sequenced in our study overlapped with ~1,800 genes; however, none of the genes that were differentially expressed in Knutie (2014) were interrogated here. Thus, we did not have the power to test whether methylation differences corresponded to changes in gene expression associated with parasitism. An updated study of transcriptomic effects of *P. downsi* on mockingbirds using RNA‐seq with alignment to the Floreana mockingbird reference genome, as well as deeper bisulfite sequencing, could provide a better comparison of gene expression and methylation patterns.

### 4.2 Effects of permethrin exposure on zebra finches

In contrast to the equivocal results in mockingbirds, there were clearer effects of permethrin exposure on the methylation of zebra finch nestlings. A higher percentage of analyzed cytosines were differentially methylated in zebra finches than mockingbirds (14.7% vs. 1.7%). In addition, the number of DMCs we observed between treatment groups was significantly more than expected between two random groups of zebra finch nestlings (Figure 3b). Thus, we are confident that the differences we observed reflect true effects of the permethrin treatment on nestling finches.

The DMCs we observed within zebra finch promoters were associated with genes involved in mitogen‐activated protein kinase (MAPK) activity, monocarboxylic acid transport, peptidyl‐threonine dephosphorylation, and endoderm development (Figure 6b). MAPKs are a diverse group of proteins that regulate cellular responses to stimuli. Pyrethroid exposure has previously been linked to effects on MAPK expression in rodents, humans, and fish (Ihara et al., 2012; Li et al., 2018; Liu et al., 2009; Zhu et al., 2020). These effects in turn have a range of downstream phenotypic consequences, including neurological symptoms in zebrafish (*Danio rerio*; Zhu et al., 2020) and endocrine disruption in mammals (Ye & Liu, 2019). MAPK signaling affects the production of hypothalamic and pituitary hormones in vertebrates (Laine et al., 2019; Li et al., 2018), which can have effects on reproduction (Laine et al., 2019; Ye & Liu, 2019). The methylation differences that we observed in zebra finches therefore mirror known physiological consequences of pyrethroid exposure in vertebrates and indicate a role for epigenetic mechanisms in mediating those changes.

Monocarboxylic acid transport genes were also associated with differential methylation in promoter regions in zebra finches (Figure 6b). Two of the six active constituents of pyrethroids are carboxylic acids (Sattelle & Yamamoto, 1988), suggesting a response in zebra finches related to processing the active compounds found in permethrin. Specifically, permethrin exposure resulted in differential methylation in the promoter of SLC16A9, which catalyzes the transport of monocarboxylates across the cell membrane. Notably, a recent genome‐wide association study in humans also found that methylation of this gene was associated with pyrethroid exposure (Furlong, 2020). These data indicate that exposure to pyrethroids results in similar epigenetic changes in diverse vertebrates; however, it remains to be seen whether these mutations reflect an adaptive detoxification response or are related to pathological effects of exposure.

The DMCs within exons were associated with genes that had other functions, including regulation of G protein‐coupled receptor protein (GPCR) signaling, regulation of nitric oxide metabolism, and response to abiotic stimulus (Figure 6c). GPCRs are a large class of signaling proteins found in both vertebrate and invertebrate genomes that regulate cell processes including cell differentiation (Sharan & Hill, 2017). Resistance to pyrethroid insecticides in *Culex quinquefasciatus* mosquitos has been linked to GPCR signaling (Li et al., 2015; Liu et al., 2007). GPCR proteins are common targets for insecticides and human therapeutics (Sharan & Hill, 2017); however, to our knowledge pyrethroids have no documented effects on vertebrate GPCR function.

### 4.3 Reconciling differences between species

Although mockingbirds were also exposed to permethrin, we did not observe the same effects on methylation that we found in zebra finches. These differences could be due to differences in exposure between captive and free‐living birds, increased variability in the natural environment, and/or biological differences between species. The nestling period for mockingbirds is shorter than for zebra finches, so mockingbirds were likely exposed to permethrin for less time. In addition, the mockingbirds in the study were free‐living and living in a hot and humid equatorial environment, whereas the zebra finches lived in an aviary inside a climate‐controlled room. Permethrin has low environmental persistence and could have been washed away by rain or degraded by sunlight in the field (Casida, 1980; Hidaka et al., 1992). Mockingbird nests are typically large (approximately 20 cm × 20 cm × 30–40 cm) and composed of layers of twigs, moss, and grass. When treating nests in the field, we typically lifted the “nest cup” (i.e., the top layer of the nest) and sprayed the middle layer underneath; *P. downsi* are typically found in the interior of the nest, and we tried to limit exposure of nestlings to the insecticide. In contrast, the zebra finch nests in the laboratory were much smaller and formed of simple piles of shredded paper, making it impossible to avoid spraying the surface of the nest. In sum, it is likely that permethrin exposure was higher in zebra finches than mockingbirds.

Natural variation in environmental conditions may also make epigenetic effects difficult to detect in free‐living mockingbirds. We included nestlings from four different years that varied in rainfall (McNew et al., 2019). We hoped that by surveying nestlings from a range of years, we could identify the most consistent effects of *P. downsi*. However, in the field there was unavoidable variation in environmental conditions and parasite intensity. The number of parasites in control nests varied because they were naturally parasitized to different degrees by *P. downsi*. Some nestlings in permethrin‐treated nests could also have been parasitized during the first 24 hr of life before the treatment was applied. As a result, while the method we used to manipulate *P. downsi* abundance is very effective and creates highly statistically significant differences in parasite abundance between treatments, variation in the timing and intensity of parasitism may have also made epigenetic effects harder to detect. These results indicate that studies in free‐living organisms require more power than studies in the laboratory to detect underlying signals of stressors on DNA methylation.

Finally, it is possible that differences in the biology of the two species could underlie the different effects of permethrin on methylation. Our in silico digestion of the genomes demonstrated that the epiGBS method was likely to target syntonic regions of each bird's genome, and there is high conservation of genome size and structure in passerines (Ellegren, 2013). Nevertheless, differences in the physiology of the two species could underlie different epigenetic responses to permethrin exposure. Future experiments exposing different bird species to pyrethroids under controlled conditions may help determine the extent to which variation in exposure, and variation between species, explains the differences in our results.

### 4.4 Applications for conservation

The widespread use of pyrethroids has raised concerns globally about nontarget effects on humans and native wildlife (Hu et al., 2019; Ye & Liu, 2019). In the Galápagos Islands, the use of permethrin for controlling *P. downsi* is contentious because of concerns about its toxicity for nestlings and native arthropods (Bulgarella & Palma, 2017; Causton & Lincango, 2014). Other studies have found sublethal effects of direct exposure of permethrin on nestling growth in the field and in captivity (Bulgarella et al., 2020; López‐Arrabé et al., 2014), and heavy exposure over multiple generations decreases fledging success of captive zebra finches (Bulgarella et al., 2020). These studies also point out that using pyrethroids to reduce parasite burden creates an “unbalanced” experiment because the insecticides themselves may have effects on nestling growth or physiology, thus underestimating the costs of parasitism and masking the effects of pyrethroids (Hund et al., 2015; López‐Arrabé et al., 2014). Our results similarly suggest that permethrin may affect developing nestlings in captivity when they are directly exposed to permethrin; however, these effects could be largely minimized in field conditions if exposure is limited. Since *P. downsi* can have devastating effects on the reproductive success of Galapagos birds, we suggest that the typically heavy costs of parasitism be weighed against the likely sublethal effects of permethrin‐based control. Nevertheless, we support the investigation of control methods that limit exposure of birds to insecticides, such as PermaCap or heat treatments (Causton & Lincango, 2014; Hund et al., 2015).

### 4.5 Effectiveness of the epiGBS approach

Bisulfite sequencing is considered the “gold standard” for assessing DNA methylation (Lea et al., 2017). This method is built on the sensitivity of unmethylated cytosines to bisulfite treatment, which are converted to uracils, while methylated cytosines are left unchanged. Unlike immuno‐precipitation methods such as MeDIP, this method recovers information about methylation of individual nucleotides and can localize epigenetic differences more precisely to genomic features (Richards et al., 2017; Robertson & Richards, 2015; Schrey et al., 2013). Whole‐genome bisulfite sequencing is often cost‐prohibitive; however, reduced‐representation methods including RRBS and epiGBS have emerged as common approaches in ecological epigenetic studies (Baerwald et al., 2016; Gawehns et al., 2020; Heckwolf et al., 2020; Hu et al., 2018; Lea et al., 2016; Mäkinen et al., 2019; Meröndun et al., 2019; van Moorsel et al., 2019). Another reason we chose epiGBS is because it does not require a reference genome, which was not yet available for the Galápagos mockingbird at the time the samples in this study were sequenced.

Although epiGBS provides an opportunity to characterize DNA methylation across a broad range of study organisms, the method is still relatively new (Sepers et al., 2019) and validation of the coverage necessary to detect different effect sizes is lacking (Boquete et al., 2020; Gawehns et al., 2020). We used simulations and sensitivity analyses to determine whether varying sample number or coverage thresholds would change our results (Table 1; Figure 3). Our results indicate that greater overall sampling depth across more samples is necessary to detect epigenetic effects, if they exist. Few other studies have used simulations or replicate experiments to generate an expected distribution of significant differences in methylation (Alvarez et al., 2020; Lea et al., 2016; Watson et al., 2021); however, we believe this approach helps distinguish true differences from artifacts.

The in silico digestion of both genomes indicated that we could expect to assay more than a million CpG positions in each genome. However, for both mockingbirds and zebra finches, we recovered only ~20–100 thousand CpG cytosines per individual. This is a much larger dataset than is typically recovered through previous methylation sequencing methods, such as MS‐AFLPs (which typically sequence hundreds of loci) or pyrosequencing (which target specific regions of the genome; Paun et al., 2019; Schrey et al., 2013; Sepers et al., 2019). Nevertheless, we sequenced only a small fraction of the estimated 10 million CpG positions in an avian genome, and a minority of our sequenced cytosines mapped to promoter regions of genes, which are thought to be most functionally relevant for methylation (Mäkinen et al., 2019; Paun et al., 2019). Increasing coverage by splitting samples over more lanes of sequencing would likely increase our ability to detect differences between groups.

Not all methylation polymorphisms are functionally relevant; further studies associating methylation differences with changes in gene expression or phenotypes should be developed (Mounger et al., 2021; Richards et al., 2017; Richards & Pigliucci, 2020). Evidence from studies in the last 10 years does not universally support that methylation regulates gene expression, and repression of transcription by methylation mechanisms might not be as widespread as previously thought (de Mendoza et al., 2020). In fact, many transcription factors show increased DNA binding affinity for methylated DNA, and gene body methylation can be associated with high expression (de Mendoza et al., 2020; Schmitz et al. 2019). More research is necessary to understand how methylation in different parts of the gene body (promoter, exon, or intron) affects gene expression, especially in nonmodel organisms (Schmitz et al., 2019). Interpretation of any significant differences is also limited by the quality of the reference genome, as well as incomplete knowledge about the biological function of genes (Paun et al., 2019). However, genomic resources for birds are rapidly increasing (Feng et al., 2020), creating more opportunities for understanding the molecular effects of environmental stressors in birds.

## 5 CONCLUSIONS

Epigenetic modifications, including DNA methylation, are potential mechanisms of rapid adaptation to a changing environment. Methylation may mediate a response to stressors, such as emerging parasites and environmental contamination, by altering gene expression and physiological processes. We tested for epigenetic effects of an invasive parasite on Galápagos mockingbirds and identified a limited number of differentially methylated cytosines between treatments. However, the number of changes we found was not more than expected by chance and we therefore cannot be confident that the differences were actually a result of either parasitism or exposure to the insecticide used to manipulate *P. downsi*. In contrast, we found clear effects of direct permethrin exposure on methylation of zebra finch nestlings. The fact that we did not identify strong methylation differences in our mockingbird dataset, despite having more individuals and slightly more coverage per individual compared with our zebra finch dataset, suggests that future epigenetic field studies should be designed to maximize statistical power. Some of the genes associated with epimutations in zebra finches were associated with known effects of pyrethroid exposure on vertebrates. Although permethrin is considered nontoxic for most vertebrates, it may have important sublethal effects on humans and wildlife and should be used judiciously.

## CONFLICT OF INTEREST

The authors declare no conflict of interest.

## AUTHORS CONTRIBUTION

**Sabrina McNew:** Conceptualization (lead); Data curation (lead); Formal analysis (lead); Funding acquisition (lead); Investigation (lead); Methodology (lead); Project administration (lead); Visualization (lead); Writing‐original draft (lead); Writing‐review & editing (lead). **Teresa Boquete:** Conceptualization (supporting); Formal analysis (supporting); Writing‐original draft (supporting); Writing‐review & editing (supporting). **Sebastian Espinoza‐Ulloa:** Resources (supporting). **Jose Andres:** Resources (supporting). **Niels Wagemaker:** Investigation (supporting). **Sarah A. Knutie:** Data curation (supporting); Funding acquisition (supporting); Investigation (supporting); Project administration (supporting); Writing‐original draft (supporting); Writing‐review & editing (supporting). **Christina Richards:** Conceptualization (supporting); Investigation (supporting); Methodology (supporting); Supervision (supporting); Writing‐original draft (supporting); Writing‐review & editing (supporting). **Dale H. Clayton:** Conceptualization (supporting); Funding acquisition (equal); Project administration (supporting); Resources (supporting); Supervision (equal); Writing‐original draft (supporting); Writing‐review & editing (supporting).

### OPEN RESEARCH BADGES

This article has earned an Open Data Badge for making publicly available the digitally‐shareable data necessary to reproduce the reported results. The data is available at https://doi.org/10.5281/zenodo.4672335.

## Supporting information

Figures S1–S4

Tables S1–S5

## Data Availability

Sequence data are deposited in the NCBI SRA database (accession PRJNA683215). Scripts used in analysis are available at github.com/smcnew/epigbs and archived on Zenodo https://doi.org/10.5281/zenodo.4562901.
