# Supplementary material for: Epigenetic effects of parasites and pesticides on captive and wild nestling birds
Source: Ecol Evol. 2021 May 3;11(12):7713–29. doi: 10.1002/ece3.7606 (PMC8216931; doi:10.1002/ece3.7606)
Supplement: Supplementary file 1 — Figures S1–S4 [file ECE3-11-7713-s002.pdf]

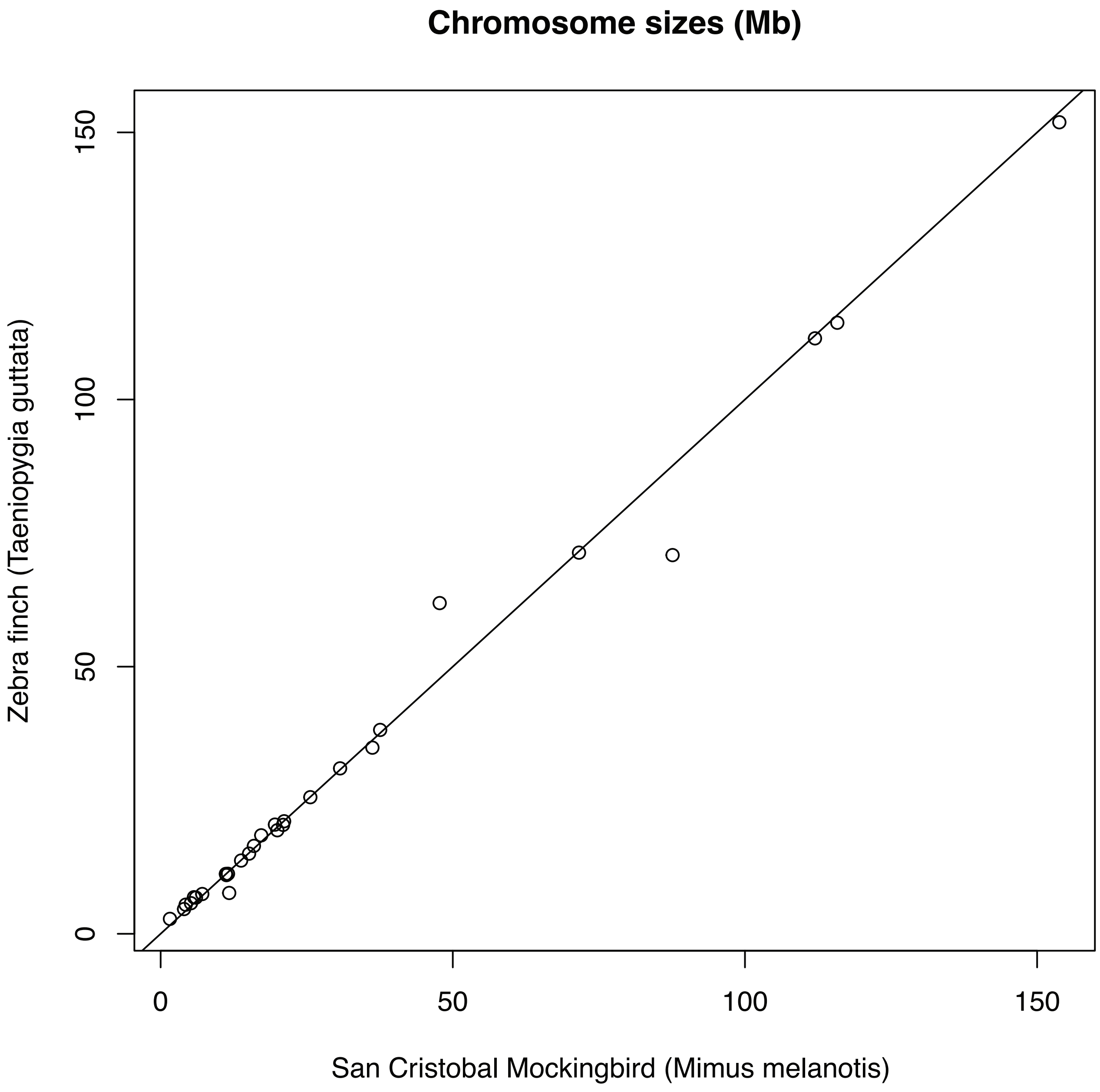

Figure S1. Comparison of the zebra finch and mockingbird reference genomes showing high correlation (r = 0.99) in chromosome sizes between species. Note that the reference genome for mockingbird was the San Cristobal Mockingbird, another endemic mockingbird from the Galapagos islands and a congener and close relative of our focal species the Galapagos mockingbird.

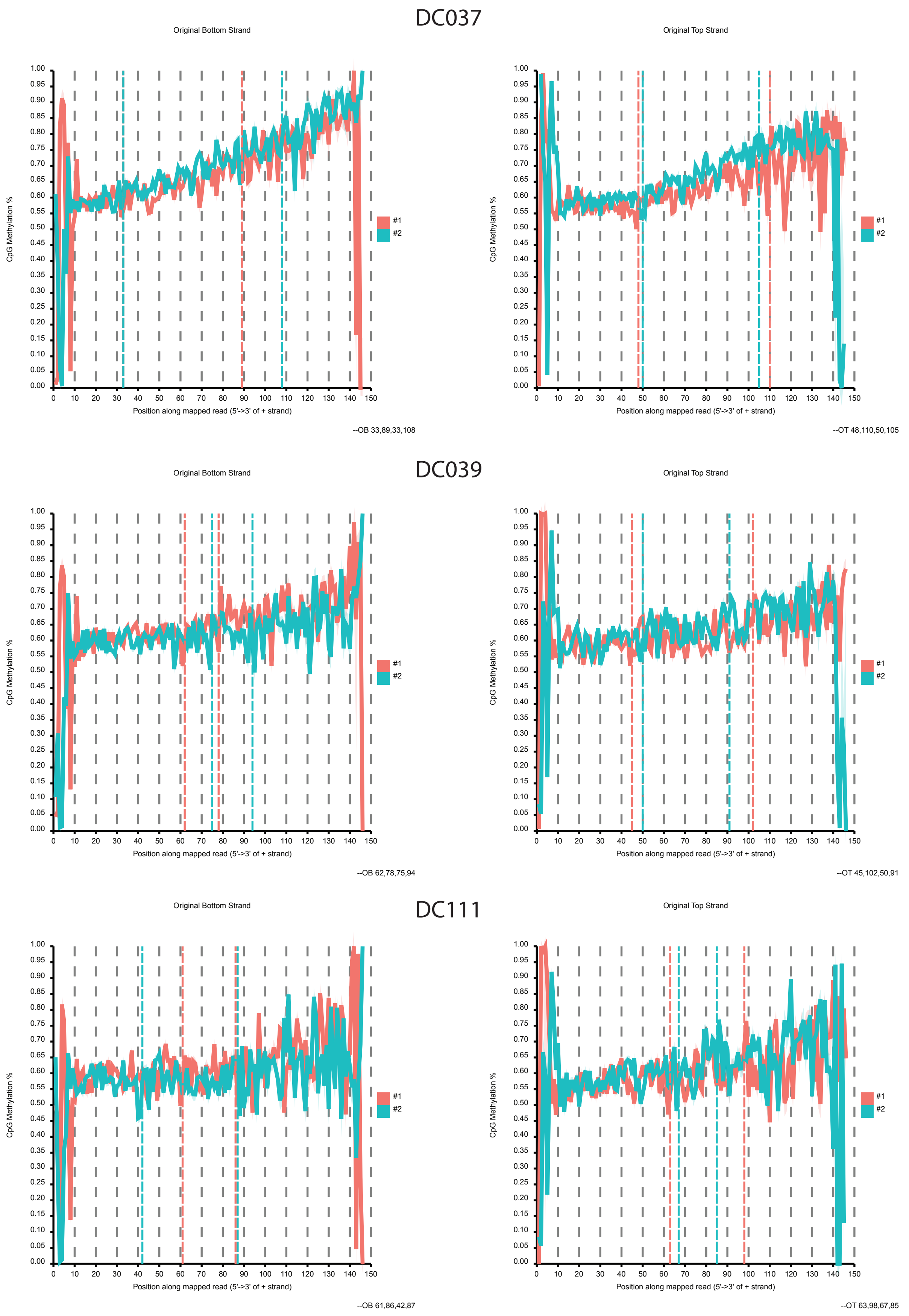

Figure S2. Methylation bias plots generated by MethylDackel showing percent methylation along the length of the read for each sample. Separate plots are shown for the top and bottom strands.

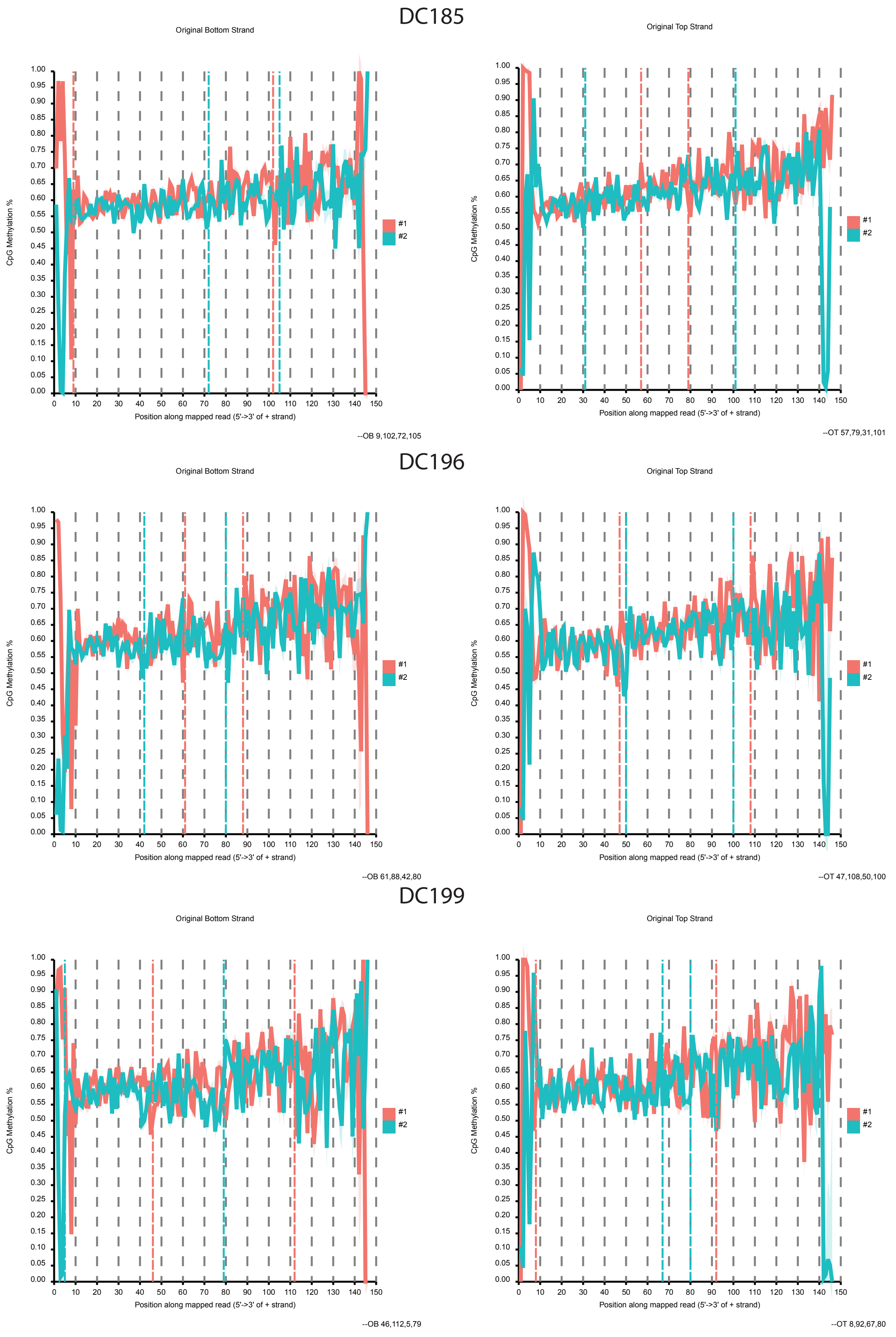

Figure S2 cont.

DC203

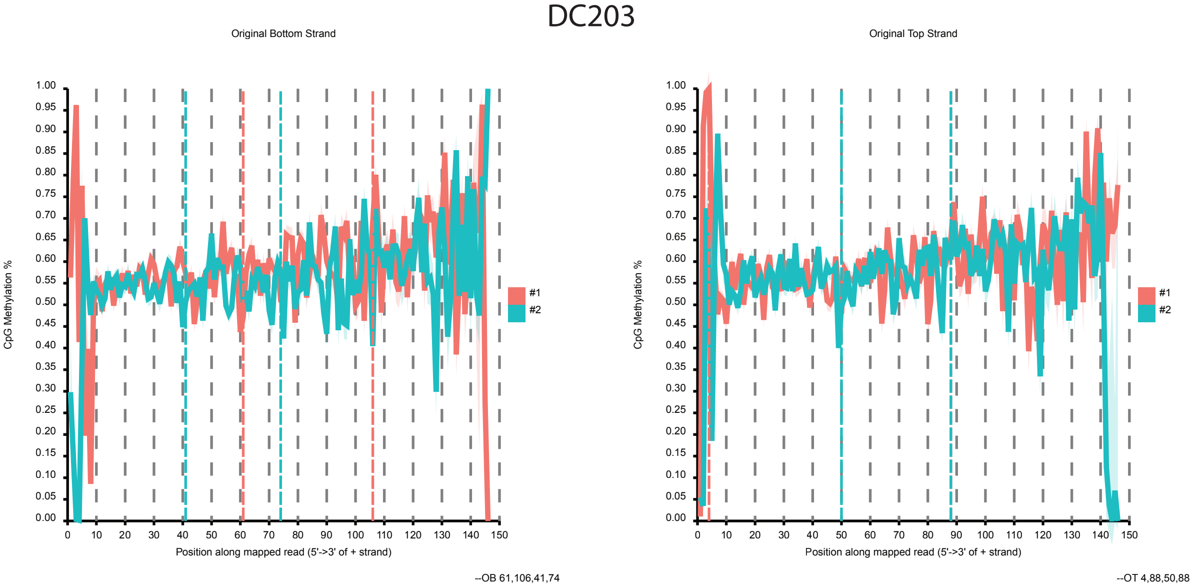

DC216

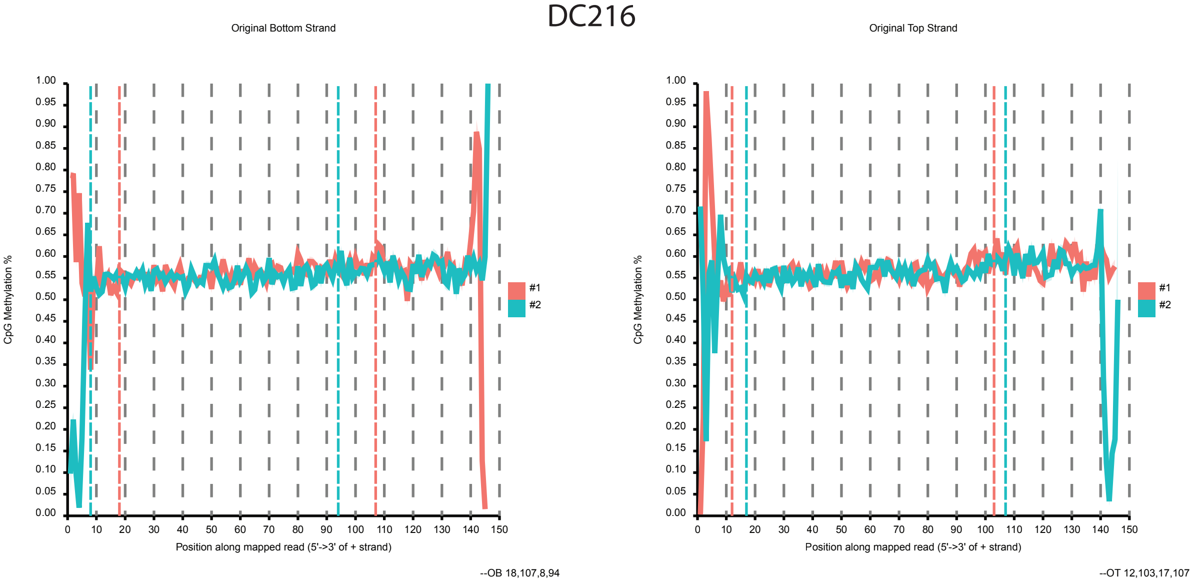

DC220

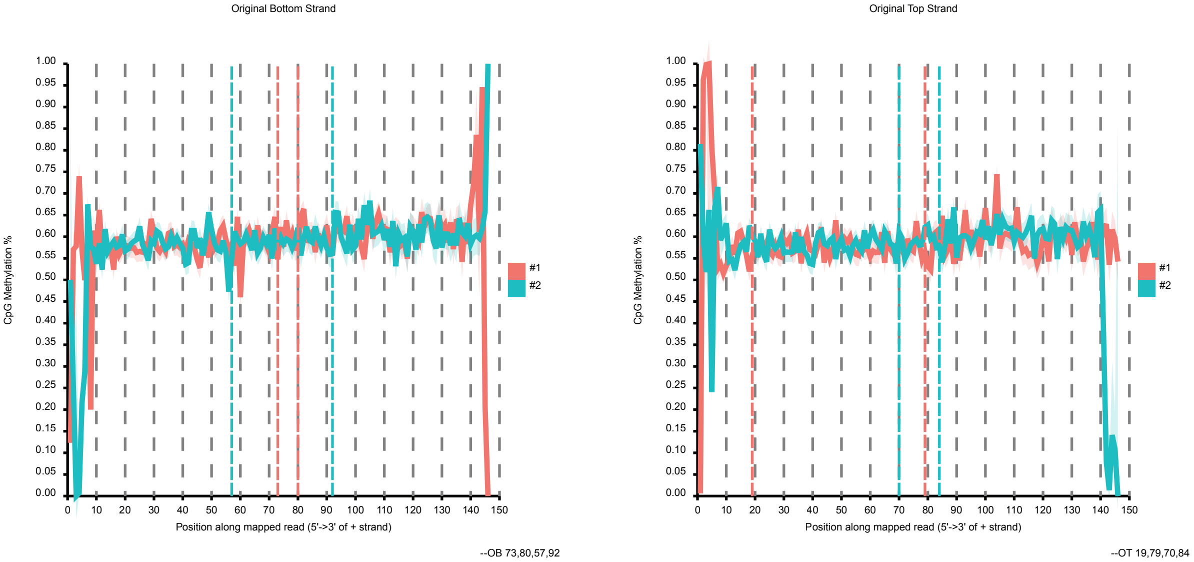

Figure S2 cont.

DC489

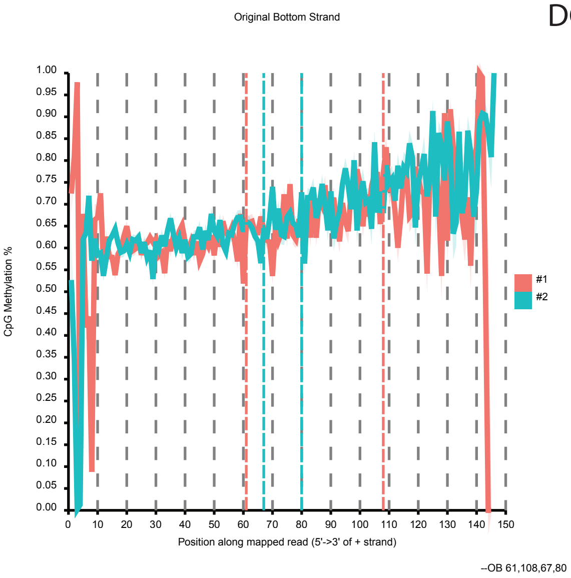

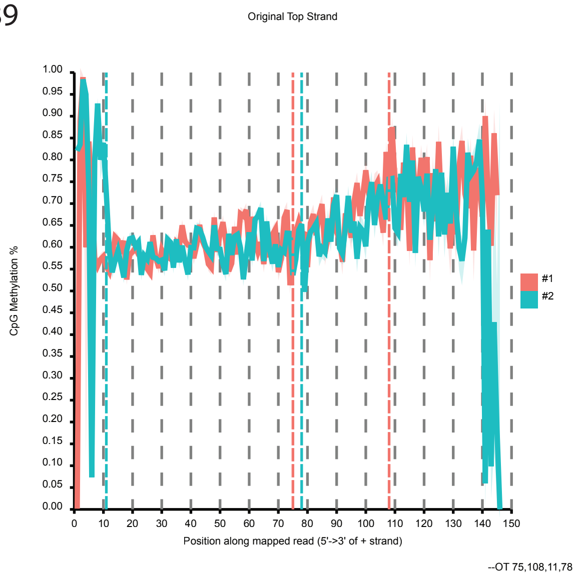

DC508

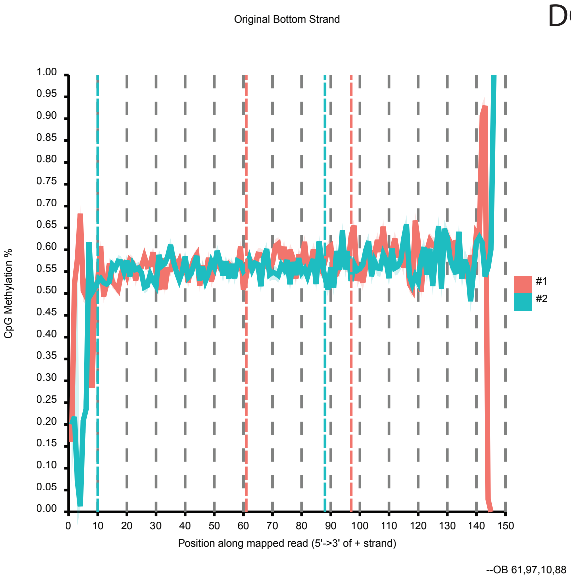

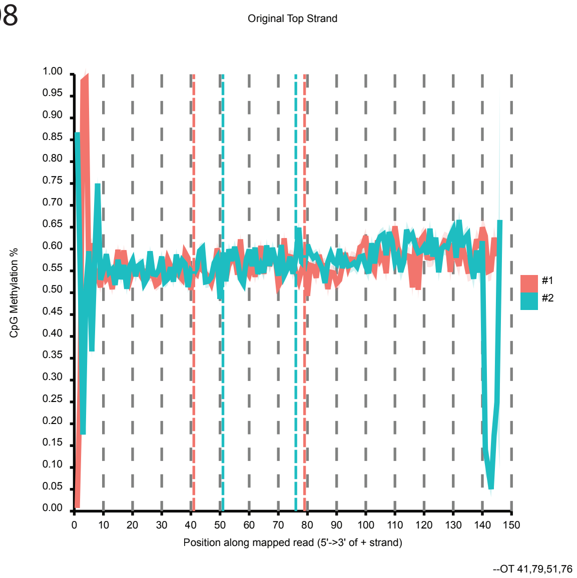

DC524

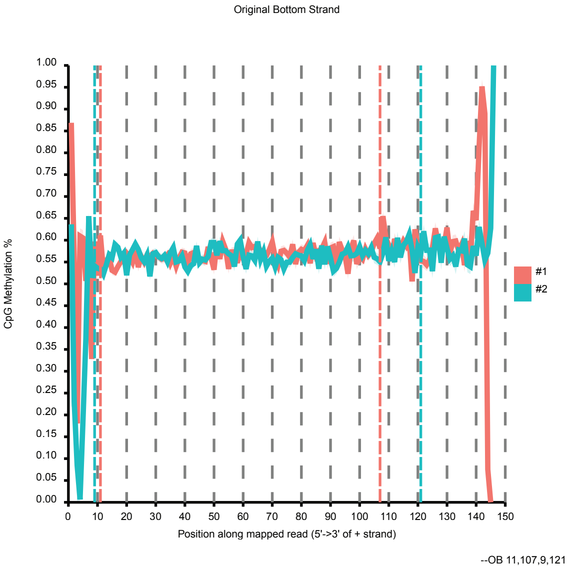

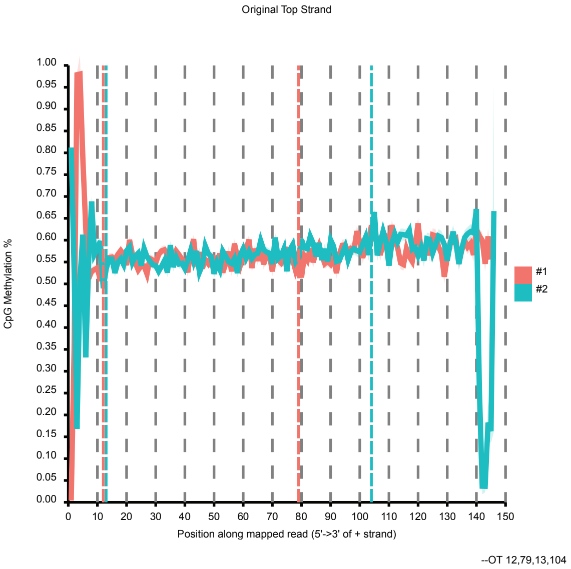

Figure S2 cont.

DC537

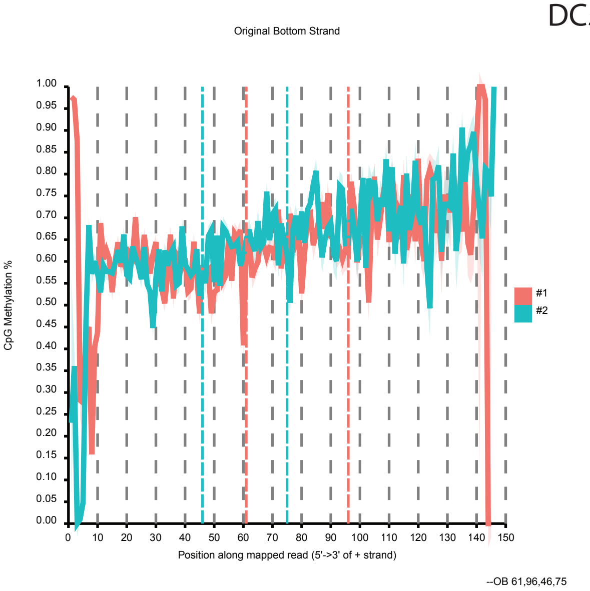

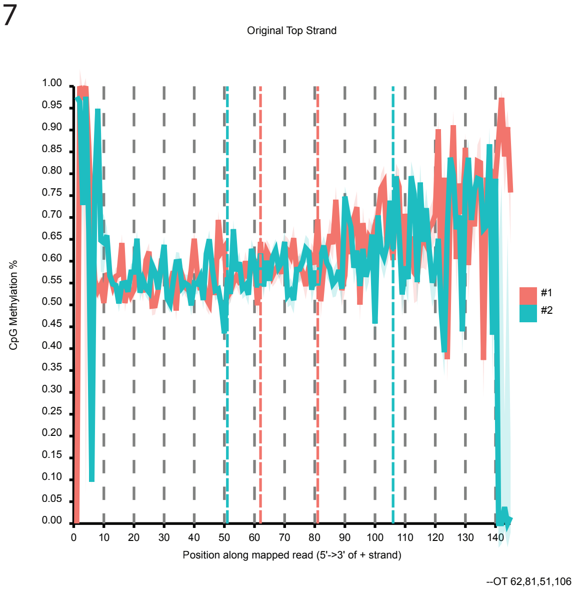

DC546

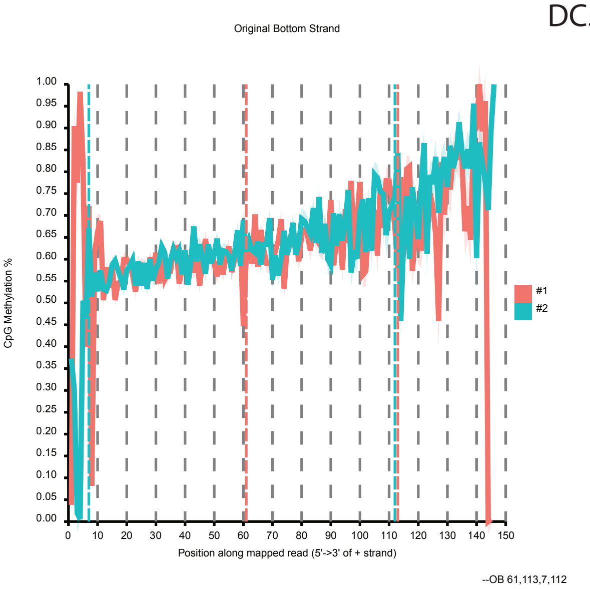

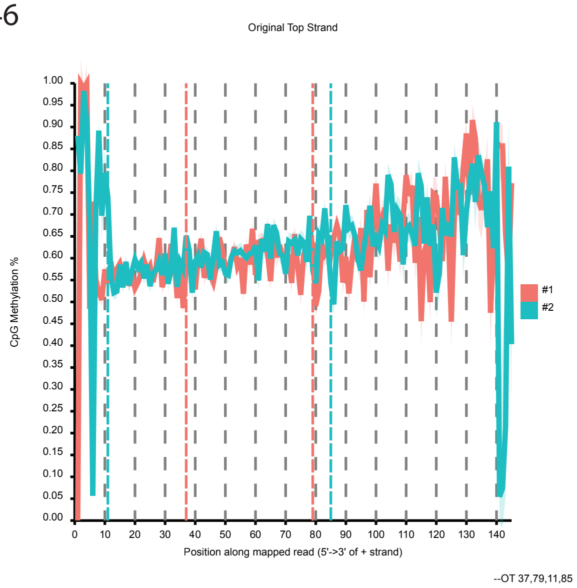

DC650

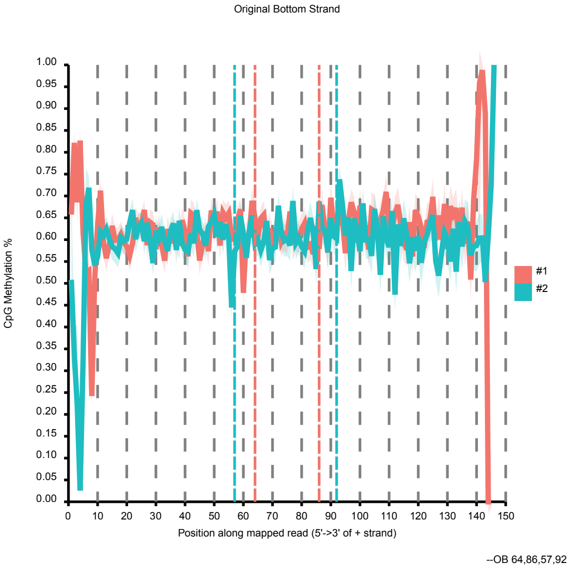

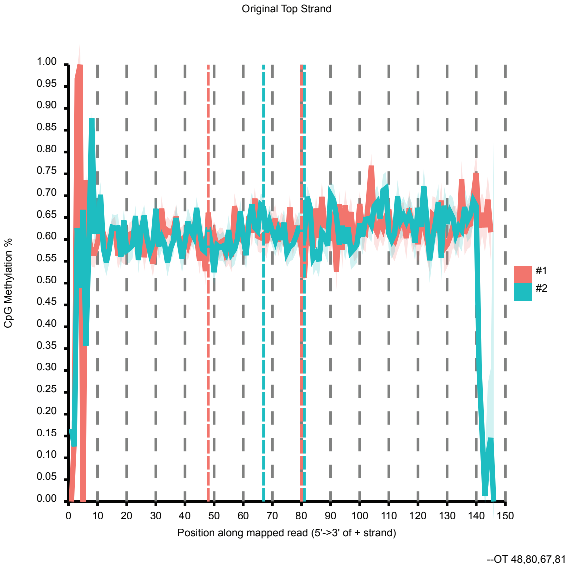

Figure S2 cont.

DC656

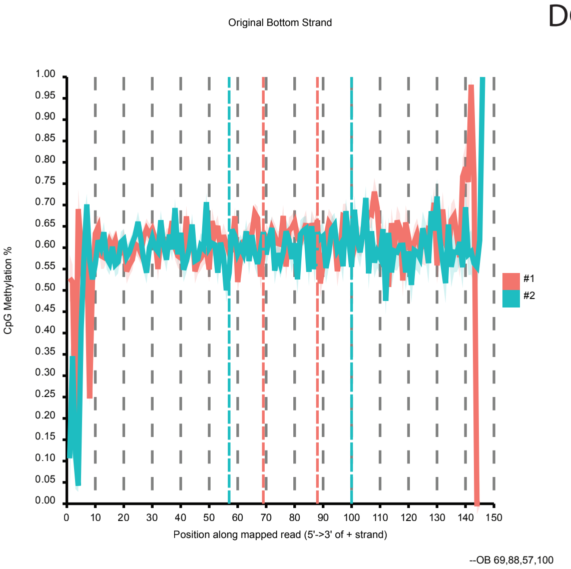

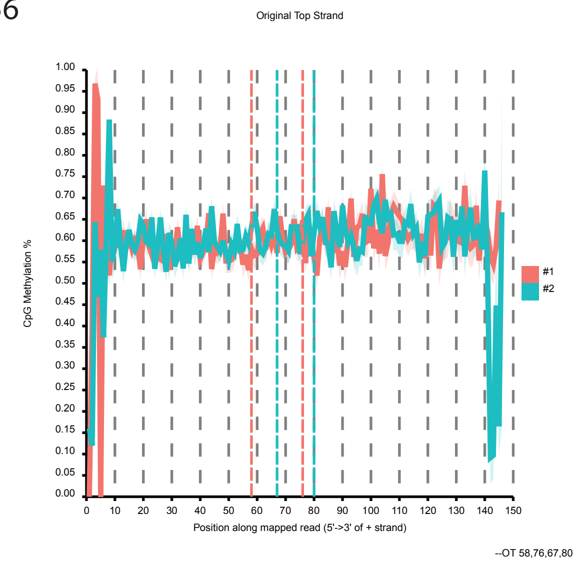

DC671

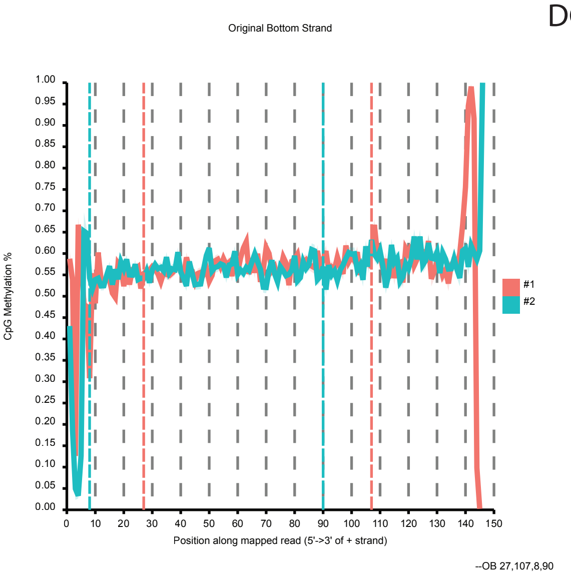

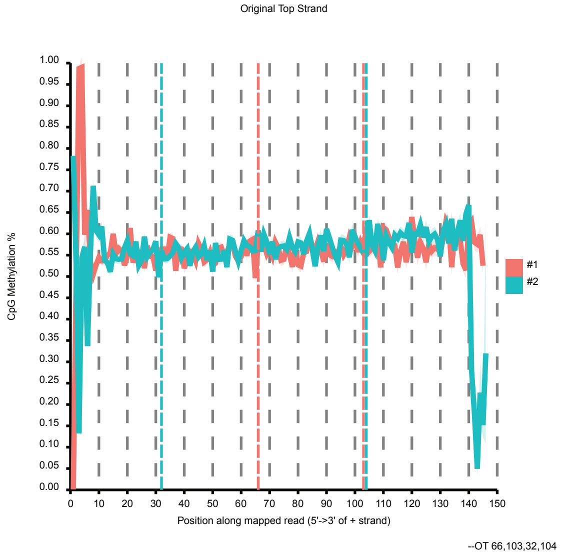

DC673

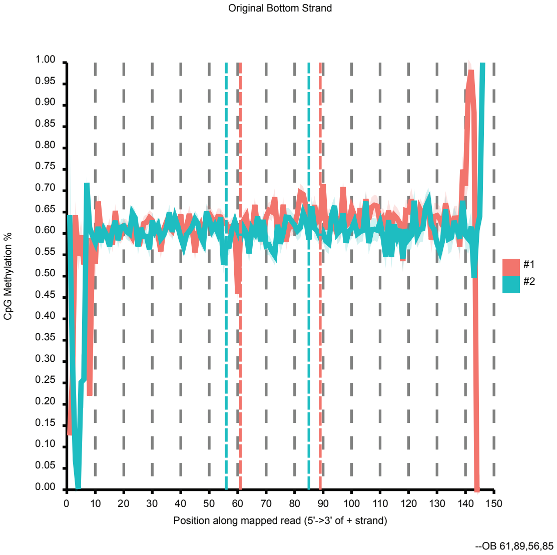

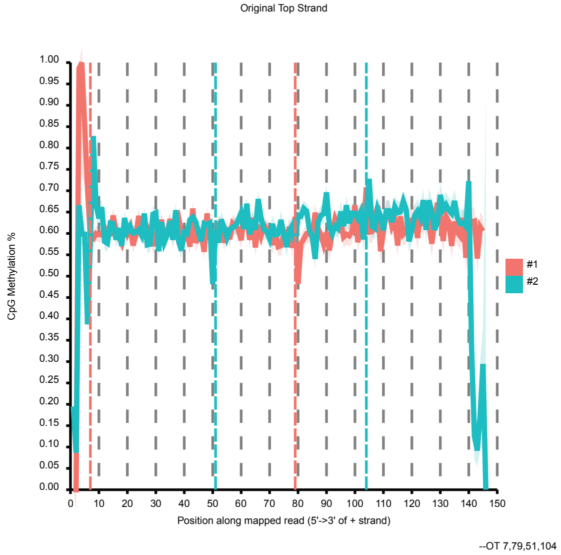

Figure S2 cont.

DC687

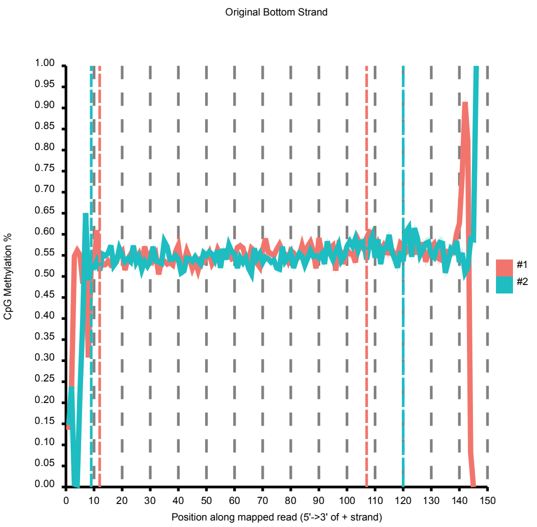

--OB 12,107,9,120

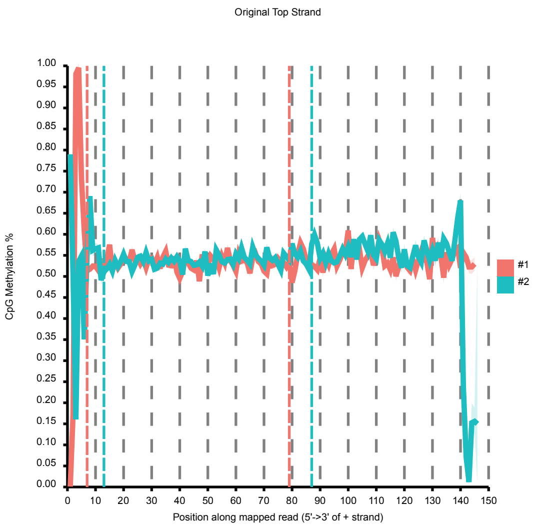

--OT 7,79,13,87

DC715

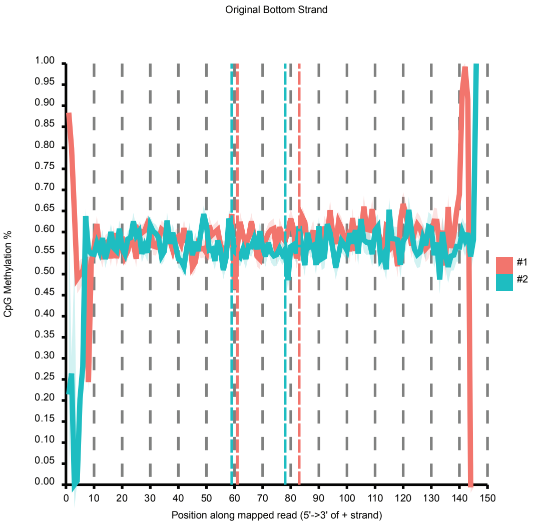

--OB 61,83,59,78

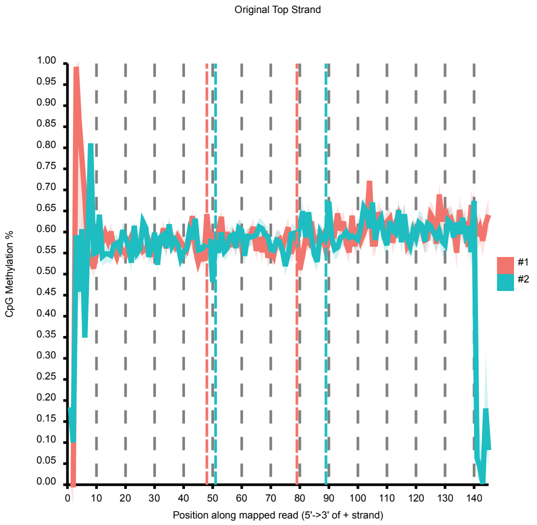

--OT 48,79,51,89

DCYY

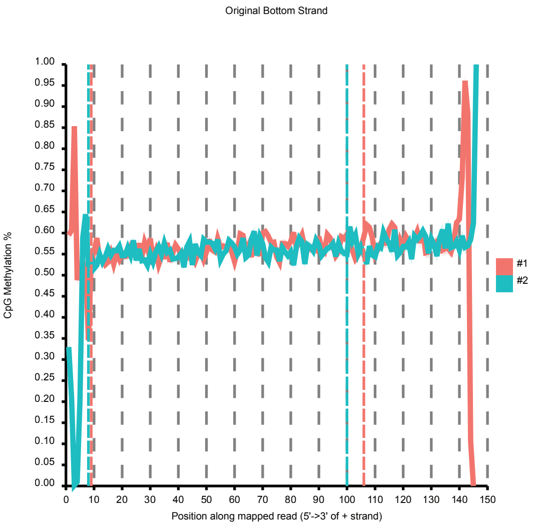

--OB 9,106,8,100

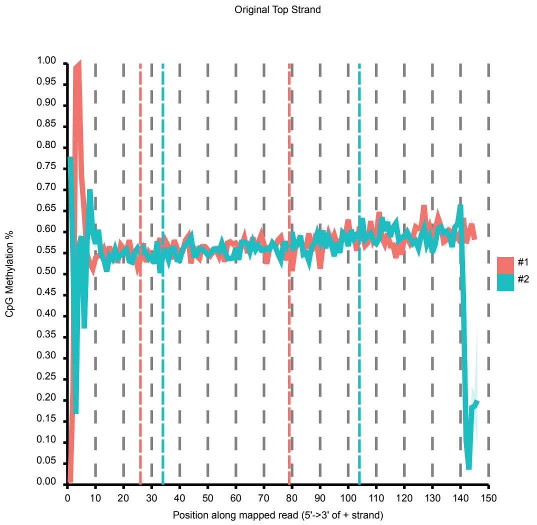

--OT 26,79,34,104

Figure S2 cont.

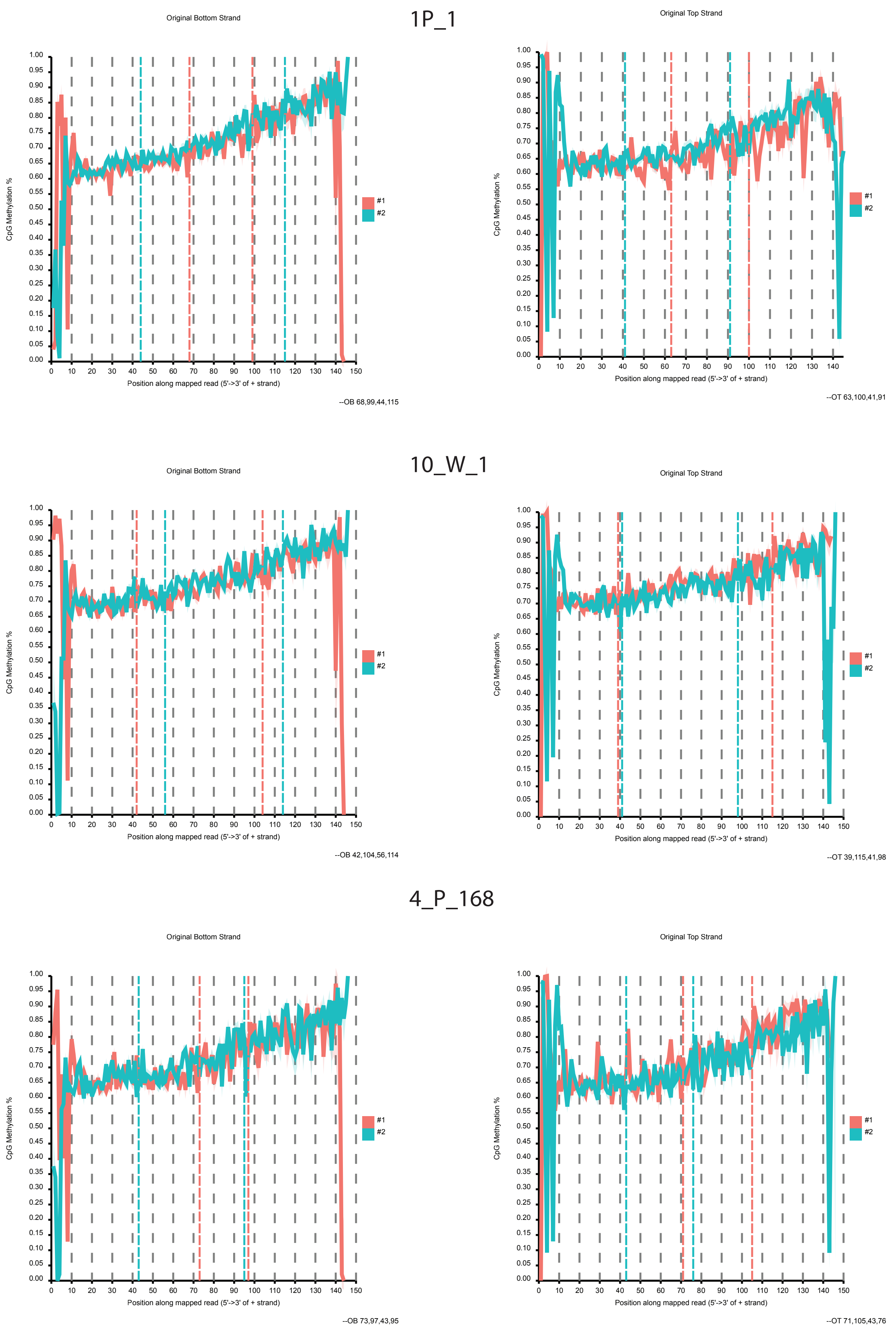

Figure S2 cont.

9_W_167

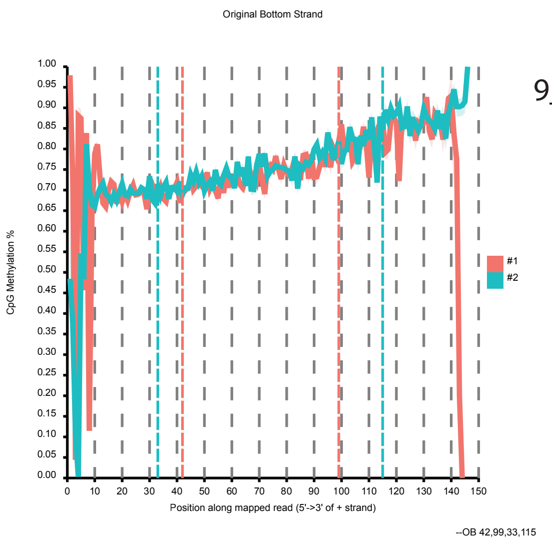

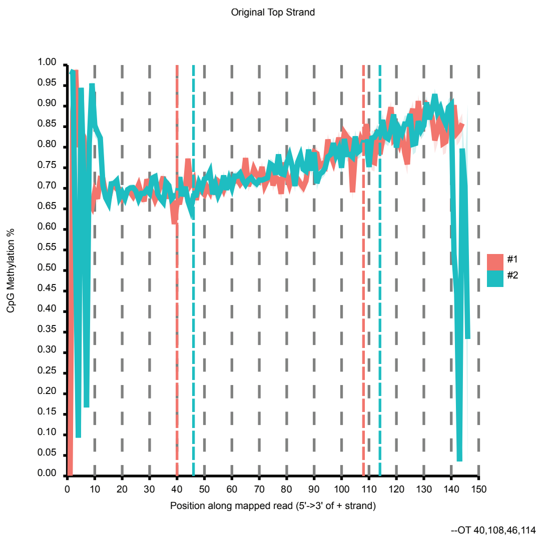

9_W_1

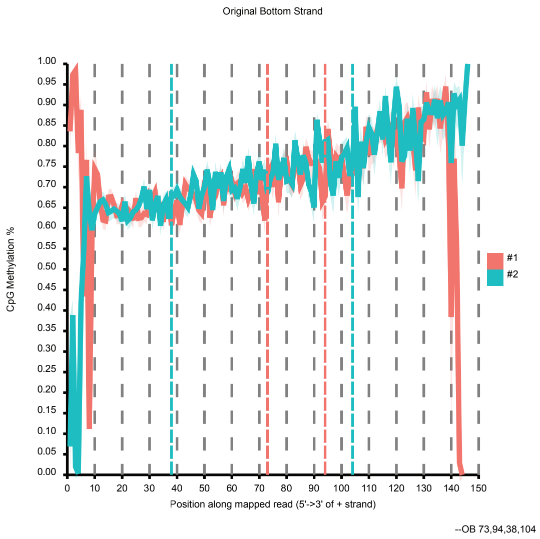

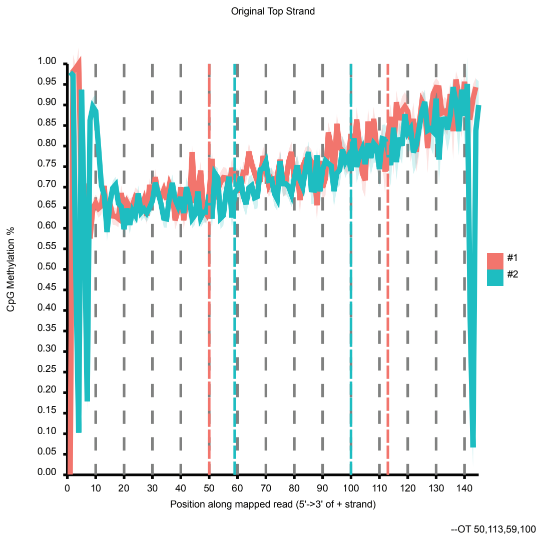

5_P_1

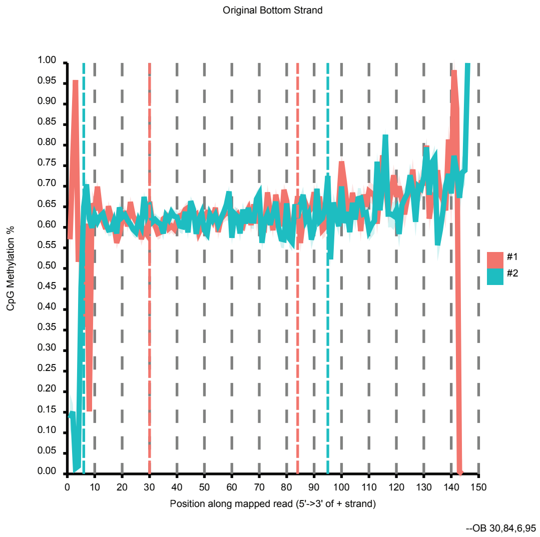

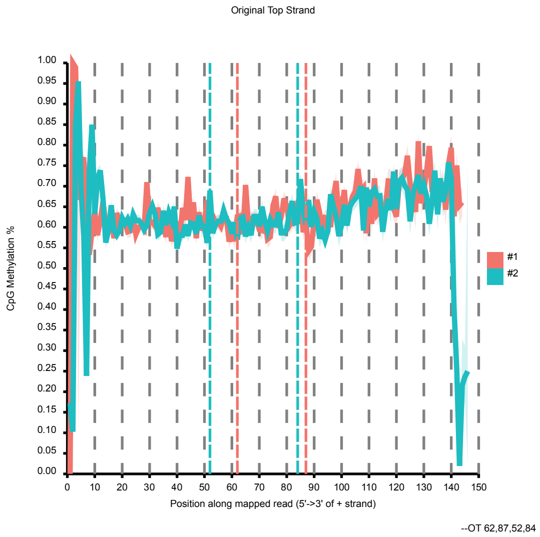

Figure S2 cont.

1P_2

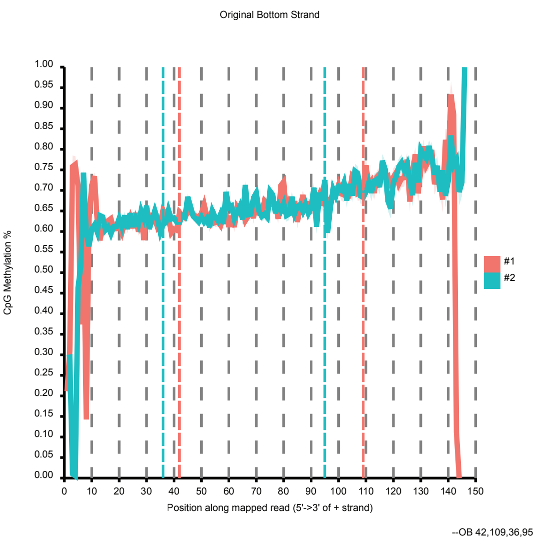

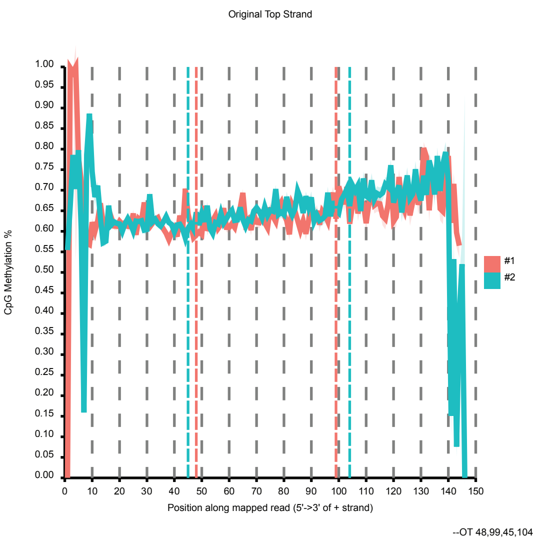

4_P_N

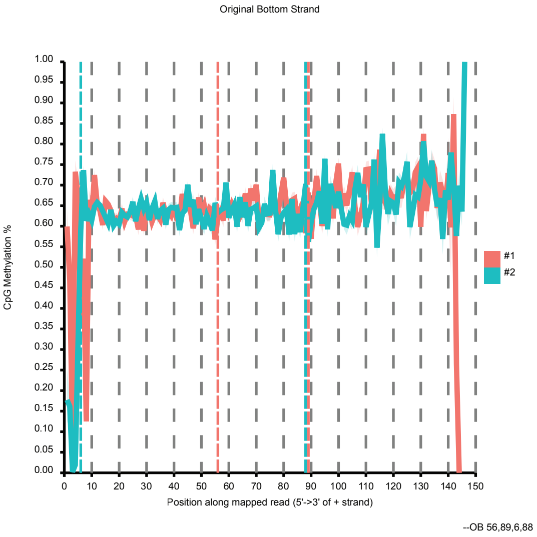

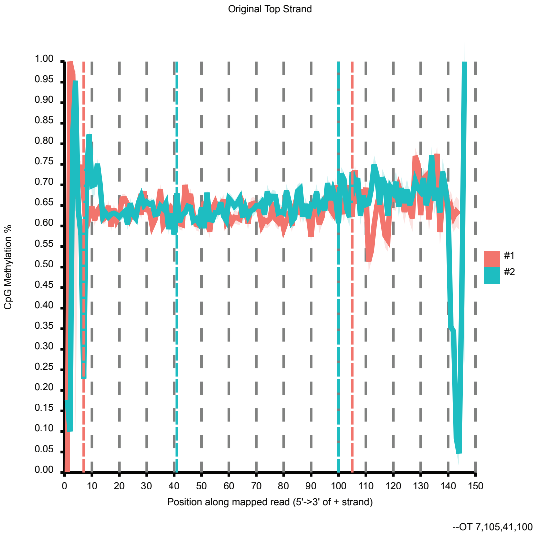

9_W_165

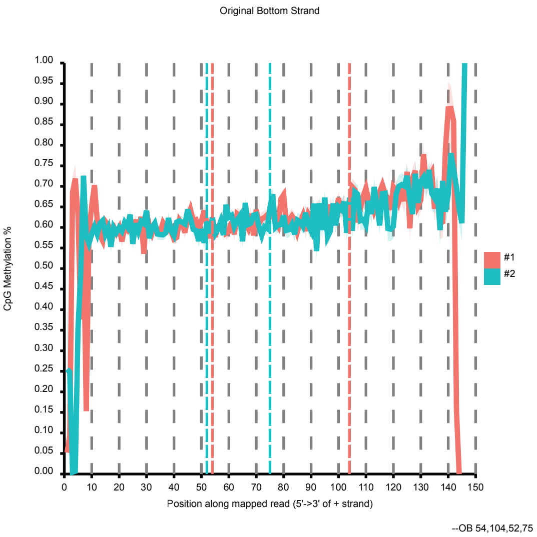

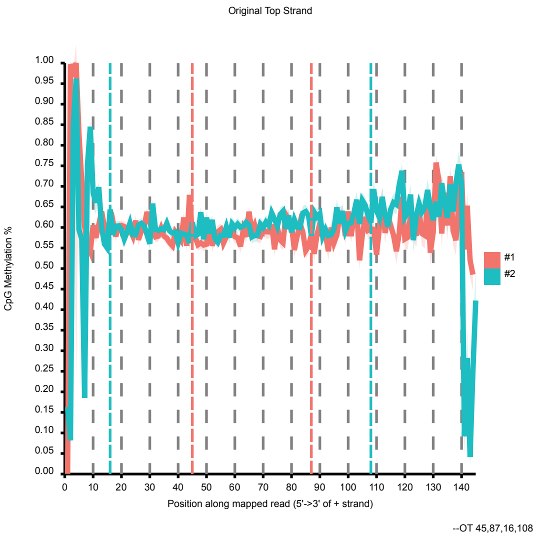

Figure S2 cont.

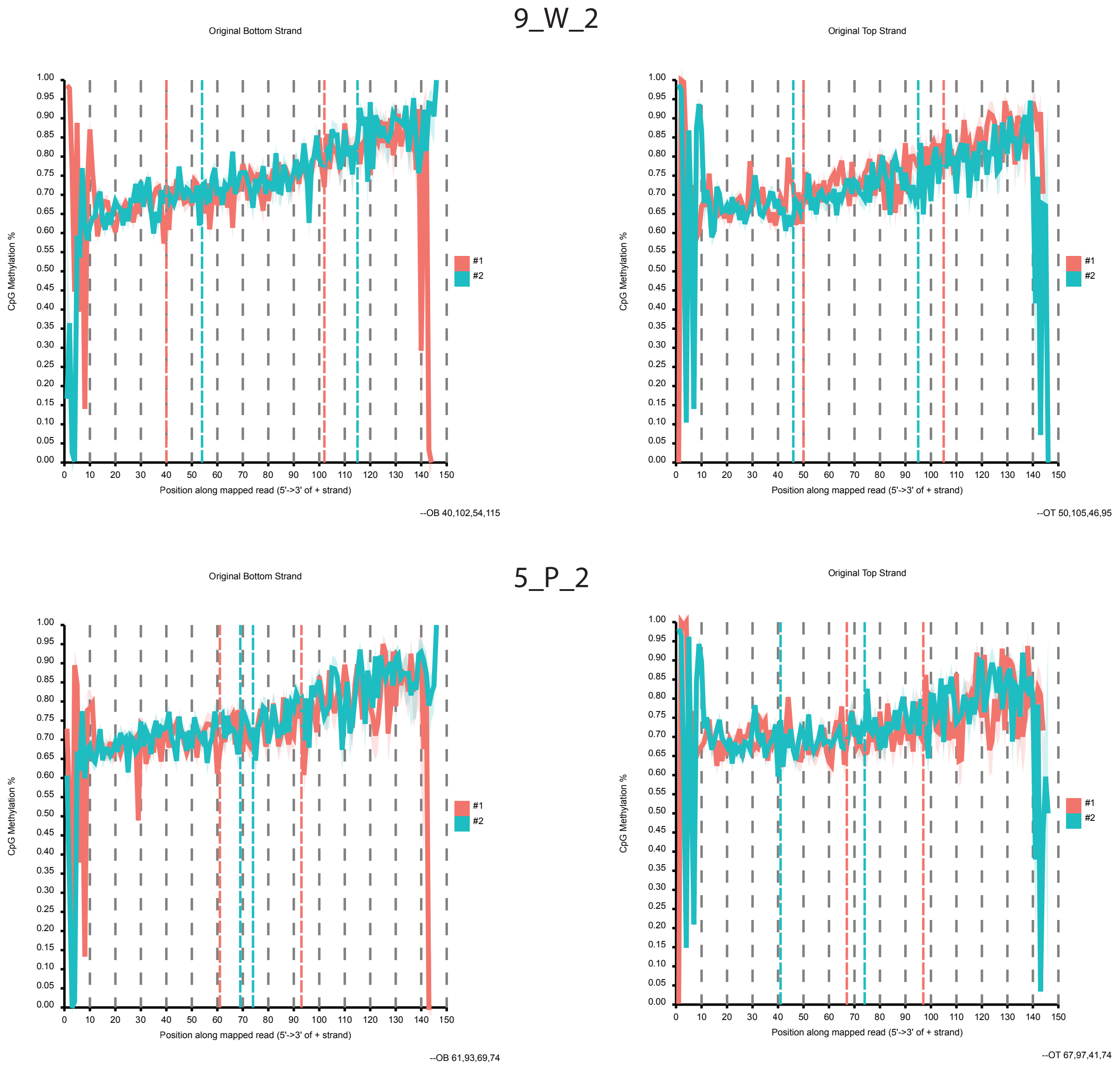

Figure S2 cont.

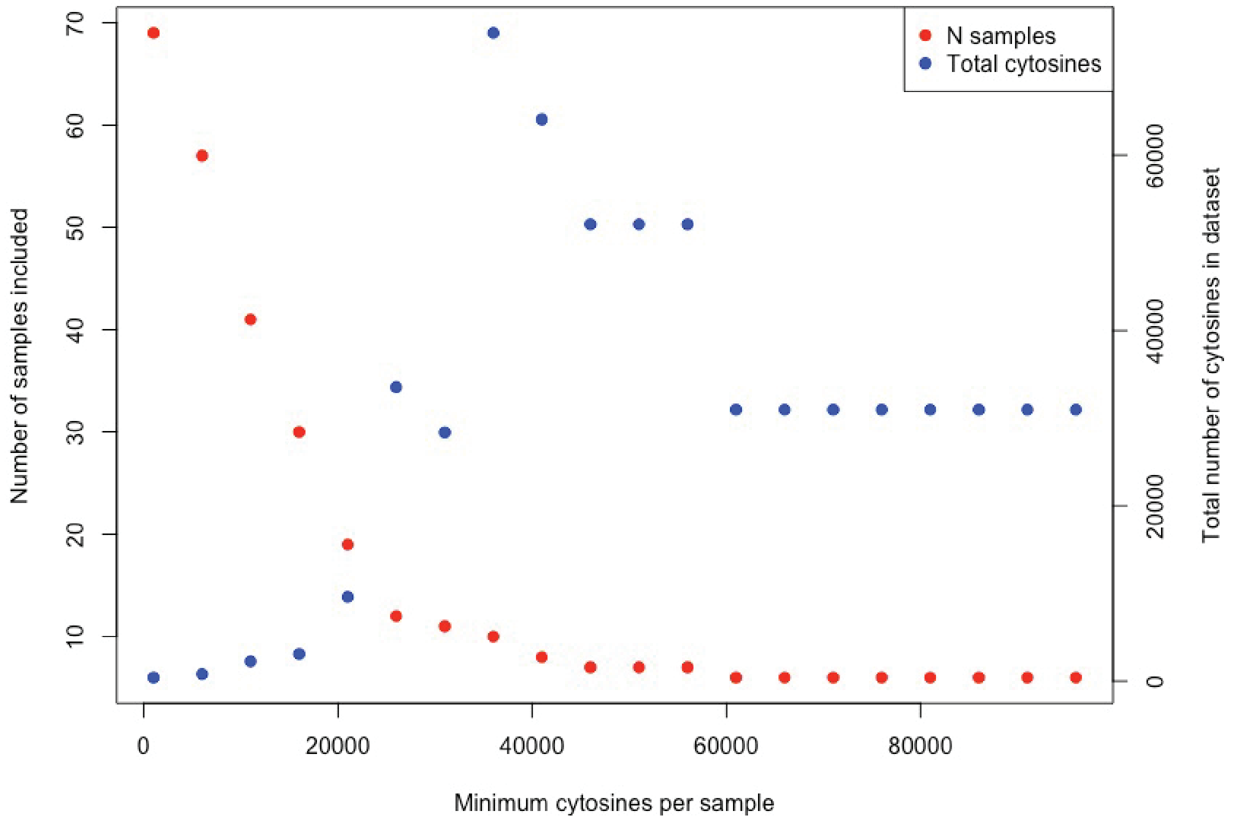

Figure S3. Analysis of coverage criteria for mockingbirds and subsequent effects on sample size and numbers of cytosines analyzed. The number of cytosines sequenced per sample ranged from 900 to 196,000. Increasing the “minimum cytosines per sample” threshold reduced overall sample size (red points). We required at least 50% of samples to have data at a cytosine in order to test that cytosine for differential methylation. Correspondingly, increasing the “minimum cytosines per sample” threshold increased the total number of cytosines analyzed, because fewer sites were dropped due to missing data (blue points). We optimized sample size and genome coverage by picking the approximate point that the curves crossed (20,000 cytosines per samples). We therefore dropped samples that had fewer than 20,000 sequenced cytosines.

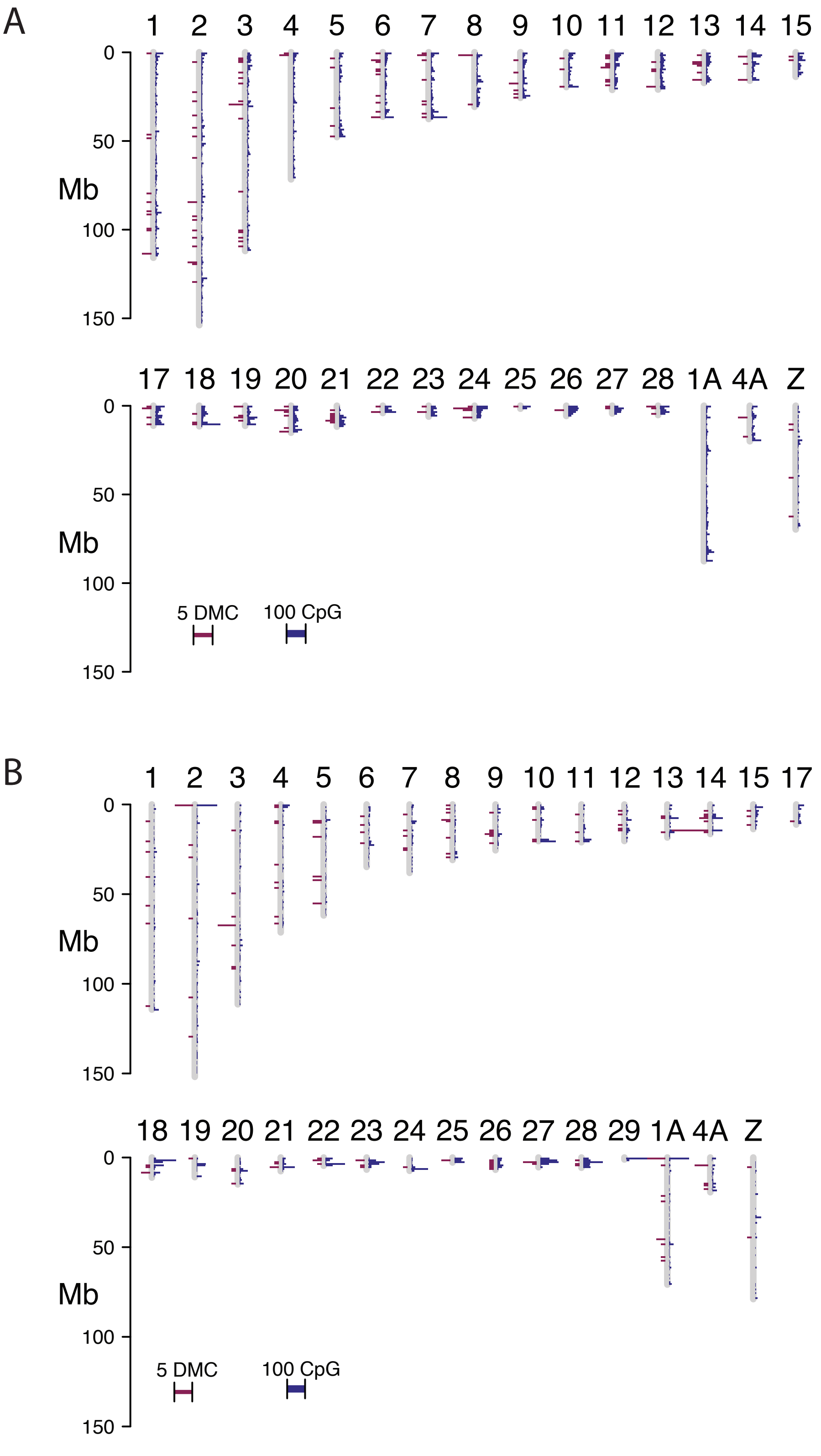

Figure S4. Chromosomal map of mockingbirds (A) and zebra finches (B) where sequenced cytosines are plotted in blue and significantly differentially methylated sites (DMCs) are shown in red. Scale bar shows the number of cytosines in that region of the chromosome. DMCs and CpGs are shown on different scales because DMCs were a smaller subset of total CpGs; the scale bars represent 5 DMCs and 100 CpGs, respectively. The thickness of scale bars has been increased for visibility.
